# Ketogenic Strategies in Neonatal Hypoxic–Ischemic Encephalopathy—The Road to Opening Up: A Scoping Review

**DOI:** 10.3390/neurolint18020024

**Published:** 2026-01-28

**Authors:** Raffaele Falsaperla, Vincenzo Sortino, Cristina Malaventura, Silvia Fanaro, Elisa Ballardini, Aloise Martina, Annamaria Sapuppo, Agnese Suppiej

**Affiliations:** 1 Department of Medical Science-Pediatrics, University of Ferrara, 44121 Ferrara, Italy; mlvcst@unife.it (C.M.); fnrslv@unife.it (S.F.); blllse@unife.it (E.B.); agnese.suppiej@unife.it (A.S.); 2 PhD Program in Innovative Innovative Technologies in Biomedical Sciences, University Kore of Enna, 94100 Enna, Italy; vincenzo.sortino@unikorestudent.it; 3 National Council of Research, Institute for Research and Biomedical Innovation (IRIB), Unit of Catania, 95123 Catania, Italy; 4 Faculty of Medicine and Surgery, University of Ferrara, 44121 Ferrara, Italy; martina.pace@edu.unife.it; 5 Unit of Pediatrics and Pediatric Emergency, Azienda Ospedaliero-Universitaria Policlinico “Rodolico-San Marco”, San Marco Hospital, University of Catania, 95123 Catania, Italy; annamaria.sapuppo@policlinico.unict.it

**Keywords:** hypoxic–ischemic encephalopathy, ketogenic diet, neonatal neuroprotection, ketone bodies

## Abstract

Background: Neonatal hypoxic–ischemic encephalopathy remains a leading cause of neonatal mortality and long-term neurodevelopmental disability worldwide. Despite the widespread adoption of therapeutic hypothermia, a substantial proportion of affected infants experience death or significant neurological impairment. Given their metabolic vulnerability, ketogenic diet strategies and ketone bodies have emerged as potential adjunctive neuroprotective interventions. This scoping review aims to critically evaluate the mechanistic rationale, preclinical evidence, and clinical feasibility of ketogenic approaches. Methods: A scoping review of the literature was conducted, including experimental and clinical studies investigating ketogenic diets, endogenous ketosis, and exogenous ketone supplementation in neonatal hypoxia–ischemia. Evidence was synthesized across mechanistic, preclinical, nutritional, and clinical domains, with particular attention to developmental context, timing of intervention, safety considerations, and translational relevance in the contest of therapeutic hypothermia. Results: Preclinical studies consistently demonstrate that ketone bodies enhance cerebral energy metabolism, support mitochondrial function, reduce excitotoxic signaling, and attenuate oxidative stress and neuroinflammation in the immature brain. Neonatal models show preferential utilization of β-hydroxybutyrate over glucose during hypoxic–ischemic stress, suggesting intrinsic metabolic advantages. Emerging evidence also supports potential long-term effects on epigenetic regulation and white matter development, although direct causal validation in neonatal HIE remains limited. Nutritional studies indicate that carefully monitored enteral and parenteral feeding is feasible in critically ill neonates, identifying a potential window for metabolic interventions. Conclusions: Ketogenic strategies represent a plausible, multimodal approach to targeting the metabolic and inflammatory sequelae of neonatal HIE. While current evidence is insufficient to support clinical implementation, this scoping review provides a hypothesis-generating framework to guide future translational research and the design of carefully controlled clinical trials in neonatal neurocritical care.

## 1. Introduction

Hypoxic–ischemic encephalopathy (HIE) remains a leading cause of neonatal mortality and long-term neuro-disability worldwide, affecting about 1.2 million newborns globally. Survivors frequently develop cerebral palsy, epilepsy, cognitive impairment, and behavioral disorders despite advances in perinatal and neonatal care [1]. Therapeutic hypothermia (TH), when initiated within 6 h of birth, represents the current standard of care for moderate-to-severe HIE. However, even in settings with optimal implementation of TH, pooled data indicate that approximately 40–50% of treated infants either die or survive with significant neurodevelopmental impairment at follow-up [2]. This persistent “residual” burden of disease has driven the search for adjunctive and combinatorial neuroprotective strategies capable of extending the benefits of hypothermia. The existing literature highlights novel adjunctive therapies as potential add-ons to hypothermia to target secondary and tertiary phases of injury beyond the initial insult [3]. From a biological standpoint, HIE is fundamentally a “metabolic catastrophe” rather than a single injurious event. The injured neonatal brain undergoes an abrupt failure of oxidative phosphorylation, followed by a prolonged secondary phase characterized by bioenergetic crisis, mitochondrial dysfunction, oxidative stress, neuroinflammation, and network instability [4]. Traditional neuroprotective approaches have largely focused on blocking the downstream pathways—such as excitotoxicity or free radical production—with limited translational success. One major reason for these failures is that HIE pathophysiology unfolds across multiple, tightly interconnected levels, including mitochondria, synapses, glial cells, the neurovascular unit, and epigenetic regulatory programs [5]. In parallel, a seemingly distinct body of evidence has developed around the ketogenic diet (KD) and ketone bodies as neuroprotective agents. The KD has a long history of clinical use in refractory epilepsy and is now increasingly studied in acute and chronic neurologic diseases [6,7]. The KD is characterized by a marked restriction of carbohydrate intake (<10%) and a high proportion of fat with adequate protein content (approximately 80–90% of total caloric intake), leading to hepatic production of ketone bodies—acetoacetate (AcAc), β-hydroxybutyrate (β-OHB), and acetone—typically within weeks of dietary initiation [8]. Both animal studies and preclinical models of ischemia suggest that KD and ketone bodies may exert beneficial effects through metabolic and signaling pathways, including energy metabolism, mitochondrial function, oxidative stress mitigation, and modulation of inflammation and apoptosis [9]. In fact, recent evidences emphasize that ketone bodies are not merely alternative energy substrates but also potent signaling molecules capable of modulating oxidative stress, neuroinflammation, synaptic transmission, and gene expression related to neuronal resilience and plasticity [10,11]. Importantly, the fetus and the newborn are physiologically “primed” for ketone utilization. In fact, during late gestation and the early postnatal period, ketone transporters and metabolic enzymes are upregulated, and ketone bodies contribute significantly to cerebral energy requirements while serving as preferred precursors for lipid synthesis, including fatty acids and cholesterol essential for brain development [12,13]. Against this background, increasing attention has been directed toward the potential use of ketogenic dietary strategies and exogenous ketone supplementation to stabilize the injured neonatal brain during and after hypoxic–ischemic insult. This comprehensive review aims to re-examine HIE from a metabolic standpoint, focusing on the mechanisms of action of the KD and ketone bodies, their neuroprotective efficacy, and issues related to safety, tolerability, and clinical feasibility in neonates, while critically appraising the available experimental and clinical evidence.

## 2. Materials and Methods

This work was designed as a scoping review and the objective was to map and synthesize the existing evidence on ketogenic strategies and ketone bodies as potential neuroprotective interventions in neonatal hypoxic–ischemic encephalopathy, to identify key concepts, knowledge gaps, and directions for future research. A literature search was performed in PubMed and Scopus using combinations of the following keywords: “hypoxic–ischemic encephalopathy”, “hypoxic ischemic brain injury”, “ketogenic diet”, “ketone bodies”, “β-hydroxybutyrate”, “nutrition” and “alimentation”. Both preclinical and clinical studies, narrative and systematic reviews, and relevant translational studies were considered. Given the exploratory nature of the topic and the limited availability of clinical trials in neonates, no restrictions were applied regarding study design. Studies were selected based on relevance to neonatal brain injury, metabolic mechanisms, and developmental neurobiology. Data were synthesized with a particular focus on mechanistic pathways, developmental considerations, and translational implications rather than quantitative effect estimates. To focus our investigation, we limited our search to the pediatric group, employing the age filter “child: birth-18 years,” and considered only English language articles published at any time. The literature search was carried out on 5 December 2025. The screening process included original research articles, case reports, and case series. Each selected article’s full text was meticulously evaluated for eligibility, quality indicators, and reliability by two independent reviewers. Discrepancies were resolved through consensus.

Studies were eligible for inclusion if they met all of the following criteria:

1. Population and developmental relevance: studies involving neonates, neonatal animal models, or developmental experimental systems relevant to the immature brain were included. Given the exploratory and translational nature of this scoping review, studies conducted in other age of life were also considered if their findings were directly applicable to neonatal brain metabolism, mitochondrial function, or neurodevelopmental trajectories.
2. Condition and pathophysiological relevance: studies addressing hypoxic–ischemic encephalopathy, perinatal hypoxia–ischemia, or closely related forms of acute neonatal brain injury were included.
3. Intervention or exposure: Eligible studies examined ketogenic strategies or ketone-related interventions, including classic or modified KDs, medium-chain triglyceride-based diets, exogenous ketone bodies (e.g., β-OHB), or specific fatty acids with documented ketone-related metabolic or signaling effects.
4. Outcomes of interest: Studies were included if they reported mechanistic, metabolic, cellular, or neurodevelopmental outcomes relevant to neuroprotection, energy metabolism, inflammation, synaptic function, or structural brain development. Both short-term and long-term outcomes were considered.
5. Study design and publication type: Experimental studies (in vivo and in vitro), observational clinical studies, and relevant narrative or systematic reviews were included to comprehensively map existing evidence. Given the absence of randomized controlled trials in neonatal HIE, no restrictions were applied based on study design.
6. Language and accessibility: Only articles published in English and available as full-text publications were included.

Studies were excluded if they met any of the following criteria:

1. Developmental irrelevance: studies conducted exclusively in adult populations or mature animal models were excluded unless the findings were explicitly discussed in relation to developmental neurobiology or neonatal brain metabolism.
2. Lack of mechanistic or translational relevance: studies focusing on ketogenic interventions without addressing mechanisms or outcomes relevant to brain injury, neurodevelopment, or cerebral metabolism were excluded.
3. Non-neurological focus: studies investigating ketogenic diets or ketone bodies solely for systemic metabolic effects without relevance to the central nervous system were excluded.
4. Irrelevant clinical conditions: studies addressing neurological conditions unrelated to neonatal brain injury or developmental vulnerability (e.g., adult neurodegenerative diseases) were excluded unless they provided fundamental mechanistic insights directly applicable to the immature brain.
5. Publication type: abstracts, conference proceedings without full-text availability, editorials, commentaries, and opinion pieces lacking original data or structured synthesis were excluded.
6. Redundant or low-informative sources: studies with insufficient methodological detail or those providing redundant information without additional mechanistic insight were excluded after full-text evaluation.

**Figure 1:**
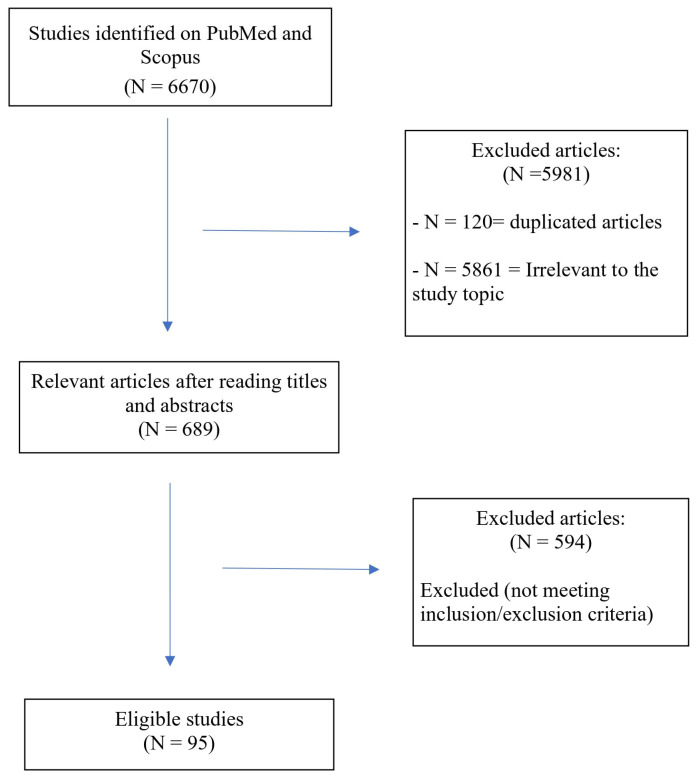
Scoping review workflow: database search, screening, and study selection.

Figure 1 provides a schematic overview of the research strategy.

## 3. Nutrition During the Acute Phase of Hypoxic–Ischemic Encephalopathy

There are currently no guidelines and/or best practices regarding feeding procedures during TH, generating a wide variability without unique clinical approach. In fact, nutritional management during the acute phase of HIE, particularly in neonates undergoing TH), has long been approached with caution due to concerns regarding gastrointestinal hypoperfusion, feeding intolerance, and necrotizing enterocolitis. However, a growing body of recent evidence suggests that early and carefully monitored nutritional support—especially enteral feeding—is both feasible and safe in this vulnerable population, challenging the traditional paradigm of prolonged nutritional restriction.

Large observational and population-based studies have provided important reassurance regarding the safety of enteral feeding during TH. Using data from the UK National Neonatal Research Database, Gale et al. demonstrated that enteral feeding during hypothermia was not associated with increased mortality or major gastrointestinal morbidity, supporting the safety of feeding practices in real-world neonatal intensive care settings [14]. Similarly, de Havilland and Hariharan, in a focused clinical analysis, concluded that enteral feeding during TH does not confer excess risk when applied prudently, reinforcing the notion that hypothermia itself should not be viewed as an absolute contraindication to enteral nutrition [15].

More recent single-center and multicenter cohort studies further refine this perspective. Molina Stornelli et al. described nutritional practices in infants with HIE undergoing TH, highlighting significant heterogeneity in the timing and modality of feeding but demonstrating that cautious advancement of enteral nutrition was not associated with increased feeding intolerance or gastrointestinal complications compared with parenteral nutrition alone [16]. Similar results were observed in a South African cohort studied by Samaai et al., in which the implementation of a standardized feeding protocol was associated with low rates of feeding intolerance during TH, supporting the broader applicability of these findings [17]. Complementing these data, Martinovski et al. similarly reported that enteral feeding during TH was well tolerated and rarely required interruption, supporting its feasibility across diverse clinical environments [18].

Importantly, emerging prospective data address not only the safety of the intervention but also the appropriate timing for its initiation. In a randomized controlled trial, Hu et al. compared early versus delayed enteral nutrition in neonates with HIE undergoing TH and demonstrated that early enteral feeding was safe and did not increase adverse events, while offering potential benefits in feeding tolerance and clinical recovery [19]. In parallel, predictive modeling studies have sought to identify infants at higher risk of feeding intolerance during TH, enabling more individualized nutritional strategies rather than blanket feeding restrictions [20].

Beyond feasibility, emerging evidence suggests that nutrition during the acute phase of HIE may actively influence brain injury evolution. Preclinical studies demonstrate that targeted nutritional supplementation can modulate neuroinflammation and lesion severity after hypoxic–ischemic injury. Brandt et al. showed that nutritional supplementation reduced lesion size and neuroinflammatory responses in a sex-dependent manner in a mouse model of perinatal HIE, highlighting nutrition as a biologically active modulator of injury rather than a purely supportive measure [21]. Similarly, Sanches et al. demonstrated dose-dependent neuroprotective effects of bovine lactoferrin following neonatal HIE, further supporting the concept that specific nutritional components can influence inflammatory and cell survival pathways in the injured immature brain [22].

Cumulatively, these data support a paradigm shift in the nutritional management of neonatal HIE. Rather than viewing nutrition as a secondary concern to be minimized during TH, contemporary evidence supports early, individualized, and metabolically informed nutritional strategies that maintain systemic homeostasis without compromising safety. This evolving framework is particularly relevant in the context of emerging metabolic interventions, as it establishes the acute phase of HIE as a window during which nutritional composition and timing may meaningfully interact with cerebral metabolism, inflammation, and recovery trajectories.

## 4. Pathophysiology of Neonatal HIE: Where Metabolic Interventions Can Act

### 4.1. Triphasic Model and Mitochondrial Vulnerability

The canonical description of neonatal HIE involves three partially overlapping phases that reflect the temporal evolution of brain injury: primary energy failure, a transient latent phase, and secondary energy failure.

Understanding this triphasic pattern is critical for identifying therapeutic windows and optimizing neuroprotective interventions:

- Primary energy failure begins during the hypoxic–ischemic insult itself, when reduced oxygen and substrate delivery force neurons away from oxidative phosphorylation. This acute phase is characterized by rapid ATP depletion, which leads to failure of ATP-dependent ion pumps, particularly the Na^+^/K^+^-ATPase. The resulting loss of ionic homeostasis causes cell depolarization, extracellular glutamate accumulation, and NMDA/AMPA receptor overactivation. The massive influx of Ca^2+^ triggers both necrotic and apoptotic cell death pathways, with early mitochondrial dysfunction and activation of calpains and other calcium-dependent proteases [23,24].
- Latent phase (approximately 6 h) is characterized by partial, deceptive metabolic recovery. During this window, mitochondrial membrane potential is restored, cerebral blood flow may normalize, and energy metabolism appears to improve. However, this apparent recovery is misleading: complex I of the electron transport chain remains vulnerable, redox balance is precarious, and multiple cell-death pathways are primed but not yet fully executed. Importantly, this is the window where TH shows the greatest benefit, as it can interrupt the progression to secondary energy failure [25]. The latent phase represents a critical therapeutic opportunity, as interventions initiated during this period may prevent or attenuate the subsequent cascade of secondary injury.
- Secondary energy failure, typically between 6 and 48 h after the insult, is associated with delayed mitochondrial collapse, persistent generation of reactive oxygen species (ROS) and reactive nitrogen species, cytochrome c release, caspase activation, ferroptosis, progressive microglial activation, and seizures. This phase is characterized by a second wave of ATP depletion, which is often more profound and sustained than the primary failure. MRI and spectroscopy studies demonstrate that the severity of secondary energy failure, as measured by lactate/N-acetyl-aspartate ratios and reduced N-acetyl-aspartate peaks, correlates strongly with adverse neurodevelopmental outcome at 18–24 months [25,26]. The mechanisms underlying secondary energy failure include ongoing mitochondrial dysfunction, accumulation of oxidative damage, inflammatory activation, and disruption of cellular calcium homeostasis.

In this context, any therapy that (a) increases the efficiency of ATP production under limited oxygen availability, (b) bypasses complex I dysfunction, (c) reduces ROS generation and oxidative damage, and (d) modulates neuroinflammation and neuronal excitability is mechanistically attractive. This profile maps closely onto ketone-based metabolism, which offers multiple points of intervention across these pathophysiological mechanisms.

The mitochondrion stands at the center of HIE pathophysiology, serving simultaneously as “victim” and “perpetrator” of injury. Mitochondrial complex I is particularly vulnerable to hypoxic–ischemic damage, and its dysfunction contributes to both inadequate ATP generation and excessive ROS production. The ability of ketone bodies to bypass complex I and enter the electron transport chain at complex II represents a fundamental metabolic advantage that may be exploited therapeutically. Additionally, ketone metabolism produces less ROS per molecule of ATP generated compared to glucose metabolism, potentially reducing oxidative stress during the recovery phase [27].

### 4.2. Developmental Peculiarities of Neonatal Brain Metabolism

The neonatal brain is not a “miniature” adult brain. From late gestation through the early postnatal period, human infants exist in a state of ‘physiological ketosis’: breast milk is high in fat (approximately 50% of calories), feeding intervals generate recurrent periods of mild fasting, and hepatic ketogenesis is robust. This metabolic state is evolutionarily conserved across mammalian species and appears to be optimized for the unique demands of brain development [28].

Monocarboxylate transporters (MCT1 and MCT2) at the blood–brain barrier and on astrocytes are highly expressed during the neonatal period, facilitating efficient ketone uptake from the circulation into the brain parenchyma. MCT1 is predominantly expressed on brain endothelial cells and astrocytic end feet, while MCT2 is more abundant on neurons. This complementary expression pattern ensures that ketones can be rapidly delivered to the sites of energy demand. The expression of these transporters’ peaks during the first postnatal weeks in rodents and remains elevated throughout human infancy, declining only as the brain matures and shifts toward greater reliance on glucose [29].

Experimental data in rodents confirm that the immature brain preferentially uses β-OHB and AcAc not only for energy production but also as carbon donors for lipid synthesis and myelin formation [30]. Studies using ^13^C-labeled tracers have demonstrated that ketone-derived carbon is incorporated into newly synthesized fatty acids, cholesterol, and structural lipids of the developing brain [31]. This dual role—as both fuel and building blocks—distinguishes ketones from glucose and highlights their unique importance during periods of rapid brain growth and myelination.

Odorcyk and colleagues provided compelling evidence for this metabolic flexibility in a rat model of neonatal hypoxia-ischemia: compared to older animals, immature rats showed higher utilization of β-OHB and better preservation of high-energy phosphates under ketotic conditions, suggesting an intrinsic ketone-based resilience of the neonatal brain. Importantly, this metabolic preference was most pronounced during the first two postnatal weeks, corresponding to the peak period of vulnerability to HIE in humans (term and near-term gestation) [32].

This developmental context is critical: it means that therapeutic enhancement of ketone availability in HIE is not an artificial imposition but an amplification of a pre-existing, physiologically relevant pathway. The neonatal brain is ‘programmed’ to use ketones, possessing the necessary enzymatic machinery, transport systems, and metabolic flexibility to shift between fuel sources. This inherent capacity may explain why neonatal brains respond more favorably to ketone-based interventions than adult brains, which have largely lost this metabolic plasticity [33].

Furthermore, the enzymes required for ketone metabolism—including succinyl-CoA:3-oxoacid CoA transferase (SCOT) and 3-hydroxybutyrate dehydrogenase—are expressed at high levels in the neonatal brain. SCOT, which catalyzes the rate-limiting step in ketone body utilization, shows developmental upregulation during the early postnatal period, ensuring that ketones can be efficiently converted to acetyl-CoA for entry into the TCA cycle. This enzymatic profile contrasts with the adult brain, where SCOT expression is lower and glucose metabolism predominates. The developmental trajectory of ketone metabolism thus creates a unique window of opportunity for ketone-based neuroprotection in the newborn period [12].

### 4.3. Ketone Bodies as Neurometabolic Modulators: Mechanistic Depth

Recent integrative reviews by Jang et al. and Makievskaya et al. have delineated a broad and interconnected spectrum of mechanisms through which ketone bodies exert neuroprotective effects in ischemic and neurodegenerative conditions. In the specific context of neonatal HIE, at least five mechanistic axes appear particularly relevant.

First, ketone bodies provide an efficient alternative energy substrate that can partially bypass impaired glycolysis and support mitochondrial ATP production during periods of glucose hypometabolism and mitochondrial dysfunction, a hallmark of the secondary energy failure following hypoxia–ischemia. By enhancing oxidative efficiency and stabilizing mitochondrial respiration, ketones may attenuate the bioenergetic collapse characteristic of the injured neonatal brain [6,10].

Second, ketone bodies—particularly β-OHB—exert direct mitochondrial effects that extend beyond energy provision. These include stabilization of the mitochondrial membrane potential, reduction in ROS production, and inhibition of mitochondrial permeability transition pore opening, thereby limiting apoptosis and necrotic cell death [34,35].

Third, ketone bodies function as signaling metabolites with potent anti-inflammatory properties. In this contest, neuroinflammation represents a central and sustained component of secondary and tertiary brain injury following neonatal HIE, characterized by prolonged activation of innate immune pathways, microglial priming, and dysregulated cytokine signaling. Within this framework, β-OHB has emerged as a potent immunometabolic modulator. Experimental studies have demonstrated that β-OHB directly inhibits activation of the NLRP3 inflammasome, a multiprotein complex critically involved in the maturation and release of interleukin-1β (IL-1β) and interleukin-18 (IL-18), key drivers of neuroinflammatory cascades after hypoxic–ischemic injury. This inhibitory effect appears to be mediated by suppression of potassium efflux and attenuation of mitochondrial ROS signaling, both of which are required for NLRP3 activation [12,36,37].

Beyond inflammasome inhibition, β-OHB modulates microglial activation states, shifting microglia away from a pro-inflammatory, cytotoxic phenotype toward a more homeostatic or reparative profile. In the neonatal brain, where microglia play dual roles in injury propagation and circuit refinement, such modulation is particularly relevant. Persistent microglial activation after HIE has been implicated not only in acute neuronal loss but also in impaired synaptic maturation, white matter injury, and long-term neurodevelopmental deficits. By dampening microglial reactivity and reducing the production of pro-inflammatory mediators such as tumor necrosis factor-α (TNF-α), interleukin-6 (IL-6), and nitric oxide, ketone bodies may mitigate both early and delayed inflammatory damage [38,39].

Importantly, these anti-inflammatory effects extend beyond classical cytokine suppression. In fact, β-OHB has been shown to influence transcriptional programs through epigenetic mechanisms, including inhibition of class I histone deacetylases, thereby downregulating genes involved in inflammatory signaling and oxidative stress while promoting expression of neuroprotective and antioxidant pathways [40]. This epigenetic impact may be especially significant in neonatal HIE, where inflammation evolves over days to weeks and contributes to tertiary injury processes such as impaired myelination, altered synaptic connectivity, and aberrant neurodevelopment.

Fourth, ketone bodies influence synaptic transmission and network excitability by modulating glutamatergic and GABAergic signaling, reducing excitotoxicity, and stabilizing neuronal networks. These effects are of particular importance in neonatal HIE, where seizures are frequent, often refractory, and independently associated with worse neurodevelopmental outcomes [41,42].

Finally, ketone bodies —particularly β-OHB—have emerged as endogenous epigenetic regulators capable of reshaping gene expression programs in the injured brain. One of the most extensively characterized mechanisms is the inhibition of class I histone deacetylases (HDACs), leading to increased histone acetylation and transcriptional activation of genes involved in cellular stress resistance and survival. In experimental models, β-OHB -mediated HDAC inhibition has been shown to upregulate antioxidant pathways, including genes regulated by the FOXO3A and Nrf2 transcription factors, thereby enhancing cellular defenses against oxidative stress—a key driver of delayed neuronal injury following HIE [43,44].

In the context of neonatal HIE, where epigenetic plasticity is particularly pronounced, such modulation of chromatin structure may have far-reaching consequences. The neonatal brain undergoes rapid developmental transitions involving synaptogenesis, axonal growth, and myelination, processes that are highly sensitive to epigenetic regulation. By influencing transcriptional programs related to neuronal survival, mitochondrial biogenesis, and synaptic remodeling, ketone bodies may promote not only acute neuroprotection but also more adaptive long-term network reorganization after injury [45,46].

Emerging evidence further suggests that β-OHB can regulate gene expression through additional epigenetic mechanisms beyond HDAC inhibition. These include direct histone modifications such as lysine β-hydroxybutyrylation, a post-translational mark associated with active transcription and metabolic state sensing. This modification has been implicated in the regulation of genes involved in energy metabolism, synaptic function, and cellular differentiation, linking metabolic availability to transcriptional control in a developmentally sensitive manner [47]. This mechanism may provide a compelling framework for understanding how ketone availability could influence long-term neurodevelopmental trajectories.

Importantly, the epigenetic actions of ketone bodies unfold over prolonged timescales, extending well beyond the acute injury window typically targeted by conventional neuroprotective strategies [48]. In neonatal HIE, where tertiary injury processes such as persistent inflammation, impaired synaptic maturation, and altered connectivity contribute to long-term neurodisability, the ability of ketone bodies to modulate gene expression programs may represent a unique therapeutic advantage. This epigenetic dimension supports the hypothesis that ketone-mediated interventions could influence not only survival but also functional recovery and neurodevelopmental outcomes, aligning acute metabolic support with longer-term plasticity and repair mechanisms [3].

From clinical and translational perspective, this multimodal mechanism of action is highly attractive for neonatal HIE, a condition characterized by temporally evolving and biologically heterogeneous injury processes that are unlikely to be mitigated by single-target interventions. Unlike traditional neuroprotective agents, ketone-based strategies have the potential to simultaneously address energy failure, inflammation, excitotoxicity, and maladaptive gene expression.

### 4.4. Mitochondrial Rescue and Oxidative Stress

Mitochondrial dysfunction represents a central and early event in the pathophysiology of HIE, driving secondary energy failure, oxidative injury, and cell death across neurons and glial populations. In this context, ketone bodies—primarily β-OHB and AcAc—offer a unique metabolic advantage by entering the TCA as acetyl-CoA via the enzyme SCOT [49]. This metabolic entry point effectively bypasses complex I of the mitochondrial respiratory chain, a major site of injury following hypoxia–ischemia and a critical source of ROS generation under pathological conditions. By delivering reducing equivalents downstream, predominantly at the level of complex II, ketone metabolism reduces electron leak, limits ROS production, and improves the ATP-to-oxygen (ATP/O_2_) efficiency of oxidative phosphorylation [50].

Beyond energetic efficiency, ketone bodies exert direct stabilizing effects on mitochondrial integrity. Experimental models of cerebral ischemia have consistently shown that KD or exogenous ketone administration preserve mitochondrial membrane potential, reduce opening of the mitochondrial permeability transition pore, and attenuate cytochrome c release, thereby limiting activation of intrinsic apoptotic pathways. These mitochondrial effects are accompanied by preservation of high-energy phosphate stores, including ATP and phosphocreatine, suggesting a sustained capacity for cellular energy homeostasis during the vulnerable reperfusion and secondary injury phases [51,52].

Evidence supporting the relevance of these mechanisms in the developing brain comes from seminal neonatal studies. In a neonatal rat model of hypoxia–ischemia, Dardzinski and colleagues demonstrated that dexamethasone pretreatment—associated with a marked elevation in circulating β-OHB levels prior to the hypoxic–ischemic insult—conferred striking neuroprotection. Treated pups exhibited minimal cortical injury and preservation of high-energy phosphate metabolites on phosphorus-31 magnetic resonance spectroscopy (^31^P-MRS), whereas untreated controls developed extensive infarction and profound energy failure. Importantly, the degree of neuroprotection closely paralleled elevations in ketone body availability, implicating ketone-mediated metabolic support as a central mechanism underlying this effect [53].

In addition to acute mitochondrial rescue, oxidative stress and ferroptosis have emerged as key contributors to delayed and tertiary brain injury in neonatal HIE, particularly affecting pre-myelinating oligodendrocytes and white matter integrity. Ketone-based interventions appear to modulate these pathways at multiple levels. As reviewed by Makievskaya and colleagues, KD and ketone supplementation reduce lipid peroxidation, enhance glutathione redox capacity, and downregulate ferroptosis-related signaling cascades in models of ischemic brain injury [6]. These effects are mechanistically linked to improved mitochondrial redox balance, decreased iron-dependent oxidative damage, and preservation of membrane lipid integrity. Given the vulnerability of oligodendrocyte progenitor cells to oxidative and ferroptotic injury in neonatal HIE, such mechanisms provide a plausible link between ketone metabolism and protection of developing white matter, with potential implications for long-term motor and cognitive outcomes [54].

Collectively, these data position ketone bodies as potent modulators of mitochondrial resilience and oxidative homeostasis in the injured neonatal brain.

### 4.5. Excitotoxicity and AMPA Receptor Modulation

Glutamate-driven excitotoxicity represents a core mechanism of neuronal injury and seizure generation in HIE, contributing both to acute neuronal death and to the propagation of pathological network activity [55]. Excessive extracellular glutamate accumulation following hypoxia–ischemia leads to sustained activation of ionotropic glutamate receptors, particularly NMDA and AMPA receptors, resulting in calcium overload, mitochondrial dysfunction, and activation of downstream cell death pathways [56]. While NMDA receptors have long been considered central mediators of excitotoxic injury, growing evidence highlights a critical role for AMPA receptor-mediated fast excitatory transmission in the amplification and synchronization of epileptiform discharges, especially in the immature brain [57].

This mechanism is particularly relevant in the neonatal period, where the balance between excitation and inhibition is developmentally skewed toward excitation. High expression of the chloride importer NKCC1 and low expression of the chloride exporter KCC2 render GABAergic transmission partially depolarizing in early life, limiting the efficacy of conventional GABAergic antiseizure medications. As a result, neonatal seizures—especially those associated with HIE—are frequently refractory to first-line therapies and may themselves exacerbate excitotoxic injury [58,59]. These developmental features underscore the need for non-GABAergic strategies that directly target excitatory transmission [60,61].

In this context, the work by Chang and colleagues represents a pivotal advance. They demonstrated that decanoic acid, the principal fatty acid component of the medium-chain triglyceride (MCT) KD, acts as a direct, non-competitive antagonist of AMPA receptors at therapeutically relevant concentrations. In hippocampal slice models, decanoic acid—but not β-OHB or AcAc—significantly reduced epileptiform discharges and inhibited AMPA receptor-mediated currents in a subunit- and voltage-dependent manner. Structural and electrophysiological analyses suggest that decanoic acid binds to the M3 transmembrane helix of the GluA2 subunit at a site distinct from that of perampanel, a clinically approved non-competitive AMPA antagonist, indicating a novel mechanism of receptor modulation [62,63].

These findings position decanoic acid as a metabolically embedded, non-GABAergic antiseizure agent within the KD, with particular relevance for neonatal seizures associated with HIE. Importantly, this mechanism bypasses the developmental limitations of GABAergic pharmacology and directly targets a key driver of pathological excitation. Rogawski et al. proposed that AMPA receptor antagonism by decanoic acid may account for a substantial component of the antiseizure efficacy of the MCT KD, independent of ketosis per se [64].

Beyond direct AMPA receptor blockade, ketogenic interventions may further rebalance excitatory–inhibitory tone through complementary metabolic mechanisms. Reduction in glycolytic flux associated with KD has been shown to decrease presynaptic glutamate release, while enhanced astrocytic metabolism may promote glutamate uptake and its conversion to glutamine and GABA, thereby reducing extracellular excitatory drive [65]. These combined effects—direct AMPA antagonism, reduced glutamate availability, and enhanced inhibitory neurotransmitter synthesis—are particularly appealing in neonatal HIE, where excitotoxicity and seizures are tightly intertwined with ongoing metabolic and inflammatory injury.

From a translational standpoint, the AMPA-modulating properties of decanoic acid suggest that specific ketogenic formulations, such as the MCT-KD or targeted fatty acid supplementation, may offer mechanistically rational adjunctive strategies for seizure control and neuroprotection in neonatal HIE. These insights further reinforce the concept that ketogenic therapies exert pleiotropic effects extending beyond ketone bodies alone, integrating lipid signaling with synaptic and network-level modulation.

### 4.6. Neuroinflammation and NLRP3 Inflammasome

Neuroinflammation is a central driver of secondary and tertiary brain injury following hypoxic damage, contributing to ongoing neuronal loss, impaired oligodendrocyte maturation, and long-term neurodevelopmental deficits [66]. Activation of resident microglia occurs rapidly after hypoxia–ischemia and persists for days to weeks, with sustained release of pro-inflammatory cytokines such as IL-1β, TNF-α, and IL-6 [67]. These mediators amplify excitotoxicity, disrupt blood–brain barrier integrity, interfere with synaptic maturation, and impair endogenous repair processes, particularly in the developing brain [68].

A growing body of evidence identifies the NLRP3 inflammasome as a key molecular hub linking metabolic stress to neuroinflammatory signaling in HIE. NLRP3 activation leads to caspase-1-dependent maturation and release of IL-1β and IL-18, cytokines strongly implicated in neonatal brain injury and seizure susceptibility [69]. In experimental models of HIE, genetic or pharmacologic inhibition of IL-1 signaling reduces infarct volume, attenuates microglial activation, and improves functional outcomes, underscoring the pathogenic relevance of this pathway [70,71].

β-OHB, the predominant circulating ketone body during ketosis, has emerged as a potent endogenous inhibitor of the NLRP3 inflammasome. In a seminal study, Youm et al. demonstrated that BHB suppresses NLRP3 activation in macrophages by preventing potassium efflux, a critical upstream trigger for inflammasome assembly. Importantly, this effect was shown to be independent of classical G-protein-coupled receptors, mitochondrial ROS, or changes in cellular energy charge, positioning β-OHB as a direct immunometabolic regulator rather than a nonspecific antioxidant [36].

These data support the concept that ketone bodies actively modulate innate immune responses within the brain, rather than merely reflecting an alternative energy substrate.

New evidences demonstrated that fasting, caloric restriction, and ketogenic interventions suppress NLRP3 inflammasome activity, reinforcing the idea that ketosis represents a distinct immunometabolic state characterized by restrained innate immune activation [72]. In the context of neonatal HIE—where inflammation is prolonged, developmentally maladaptive, and tightly coupled to ongoing tissue injury—such modulation is particularly attractive.

Although direct experimental data on NLRP3 inhibition by ketogenic strategies in neonatal hypoxia–ischemia models remain limited, the mechanistic plausibility is strong. Wood et al. explicitly highlight NLRP3 suppression and microglial polarization toward a reparative phenotype as major candidate mechanisms underlying ketone-mediated neuroprotection in the injured newborn [12]. Importantly, modulation of neuroinflammation by ketone bodies may complement therapeutic hypothermia, which only partially attenuates inflammatory cascades and has limited impact on delayed microglial activation.

Taken together, these findings suggest that ketone bodies—and β-OHB in particular—may mitigate one of the most persistent and therapeutically challenging components of HIE pathology. By dampening NLRP3-driven cytokine release and reshaping microglial responses, ketogenic strategies could potentially reduce secondary injury, limit seizure–inflammation feedback loops, and improve long-term neurodevelopmental trajectories beyond the acute phase of hypoxia–ischemia.

### 4.7. Epigenetic Regulation and Long-Term Programming

Beyond their acute metabolic and anti-inflammatory actions, ketone bodies—particularly β-OHB—exert powerful effects on epigenetic regulation, positioning ketogenic strategies as potential modulators of long-term brain development after HIE [43]. β-OHB has been identified as an endogenous inhibitor of class I and class IIa HDACs, leading to increased histone acetylation and broad changes in gene transcription. This mechanism directly links cellular metabolic state to chromatin accessibility and transcriptional programs governing stress resistance, neuronal survival, and synaptic plasticity [37,73,74].

Jang et al. reported that ketone exposure upregulates a network of genes involved in oxidative stress resistance and mitochondrial homeostasis, including FOXO3a, superoxide dismutase, and catalase. Activation of FOXO transcription factors promotes antioxidant defense, autophagy, and DNA repair pathways, while enhanced expression of mitochondrial antioxidant enzymes improves resilience to secondary energy failure and oxidative injury—key drivers of delayed neuronal loss in HIE [10].

In addition to regulating stress-response genes, ketone-mediated HDAC inhibition has implications for synaptic plasticity and circuit maturation. Increased histone acetylation has been associated with enhanced expression of neurotrophic factors, synaptic scaffolding proteins, and activity-dependent plasticity genes, all of which are critical for adaptive rewiring after injury [75]. In the context of neonatal HIE—where synaptic networks are actively forming—such modulation may influence the balance between maladaptive hyperexcitability and functional recovery.

The relevance of these findings is amplified in the perinatal period, a developmental window characterized by heightened epigenetic plasticity. During late gestation and early postnatal life, chromatin remodeling plays a central role in regulating oligodendrocyte maturation, interneuron differentiation, synaptic pruning, and myelination. Hypoxic–ischemic injury disrupts these tightly regulated programs, leading to long-lasting alterations in connectivity and white matter integrity [76]. In this context, the HDAC-modulating properties of ketone bodies raise the intriguing possibility that ketogenic strategies may not only attenuate acute injury but also influence long-term neurodevelopmental trajectories after HIE by reshaping epigenetic programming. Although these epigenetic mechanisms are supported by robust experimental data in experimental models of brain injury and development, direct causal evidence in neonatal hypoxic–ischemic encephalopathy remains limited. Therefore, epigenetic modulation should be regarded as a promising but still emerging mechanism in this specific context.

From a translational perspective, this epigenetic dimension suggests that ketone-mediated neuroprotection may extend well beyond the acute and subacute phases of hypoxia–ischemia.

### 4.8. Myelination, White Matter Integrity and Network Maturation

White matter injury is a hallmark of neonatal HIE and represents a major substrate of long-term motor, cognitive, and behavioral impairments [77]. Beyond focal neuronal loss, hypoxia–ischemia disrupts the maturation of pre-oligodendrocytes, the predominant oligodendroglial population in the perinatal brain, which are exquisitely vulnerable to oxidative stress, excitotoxicity, and inflammation. Damage or arrested differentiation of these cells results in diffuse hypomyelination and impaired network connectivity, even in infants who escape large cortical infarctions [78,79].

Ketone bodies occupy a unique position at the intersection of energy metabolism and lipid biosynthesis, both of which are critical for myelin formation. During normal development, ketones are preferential substrates for the synthesis of fatty acids and cholesterol, essential structural components of myelin membranes [80,81]. Of interest, ketone uptake and utilization are physiologically upregulated, and ketones contribute not only to cerebral energy supply but also to the rapid expansion of myelinating white matter tracts [82]. In the context of HIE, where endogenous substrate availability and mitochondrial function are compromised, ketone-based metabolic support may therefore be particularly relevant for sustaining myelin synthesis during a vulnerable developmental window.

In addition to serving as biosynthetic precursors, ketone bodies may indirectly protect white matter by stabilizing mitochondrial function and reducing oxidative and inflammatory stress within oligodendrocyte lineage cells. Preservation of mitochondrial membrane potential, improved redox balance, and attenuation of microglial-derived cytokines—all documented effects of ketogenic interventions—are highly relevant given the sensitivity of pre-oligodendrocytes to mitochondrial dysfunction and lipid peroxidation [83]. These mechanisms provide a plausible link between ketone metabolism and reduced diffuse white matter injury after hypoxia–ischemia.

While direct data in neonatal hypoxia–ischemia models remain limited, parallel findings from models of perinatal inflammation and metabolic stress support the notion that alternative energy sources availability favors myelin-related gene expression and structural integrity of white matter tracts [84].

Importantly, white matter integrity is tightly linked to network maturation and higher-order brain function. Disrupted myelination alters conduction velocity, impairs synchronization across distributed networks, and interferes with activity-dependent circuit refinement [85]. In HIE survivors, these alterations manifest as deficits in motor coordination, executive function, attention, and processing speed, even in the absence of severe motor disability [86]. By supporting oligodendrocyte survival and maturation, ketone-based interventions may therefore exert downstream effects on large-scale network organization and long-term neurodevelopmental outcomes.

Taken together, these observations suggest that ketogenic strategies may be uniquely suited to address a critical and often underappreciated component of HIE pathology: diffuse white matter injury and impaired network maturation. It should be emphasized that, while the biological plausibility linking ketone metabolism to white matter protection is strong, direct demonstration of ketone-mediated myelin rescue in neonatal HIE models is still lacking. Consequently, effects on myelination and network maturation should be interpreted as putative longer-term mechanisms requiring targeted experimental validation.

### 4.9. Preclinical Evidence in Neonatal Hypoxia–Ischemia

Preclinical evidence supporting the use of ketogenic strategies in neonatal HIE brain injury has progressively accumulated, providing a coherent mechanistic and developmental rationale for ketone-based neuroprotection. As summarized by Zhou et al., a central premise emerging from animal studies is that the immature brain is metabolically primed to utilize ketone bodies as alternative fuels, particularly under conditions of glucose deprivation and mitochondrial stress typical of hypoxia–ischemia. Classic developmental studies demonstrated that during the neonatal period ketone bodies contribute substantially to cerebral energy metabolism and lipid synthesis, supporting both ATP generation and structural brain development [87,88]. This intrinsic metabolic flexibility suggests that ketosis may be especially relevant in the context of neonatal HIE, where failure of oxidative phosphorylation and impaired glucose utilization are early and sustained features of injury.

Direct experimental evidence for ketone-mediated neuroprotection in neonatal HIE was provided showing that β-OHB attenuated brain injury in neonatal animal models by alleviation of neuronal apoptosis, reducing oxidative stress and inflammation [89,90]. These findings provided one of the earliest demonstrations that augmenting ketone metabolism can directly stabilize cerebral bioenergetics in the immature brain during hypoxic–ischemic stress.

Subsequent studies in juvenile and developmentally immature models of cerebral ischemia and traumatic brain injury have reinforced and extended these observations, supporting the biological plausibility of ketogenic interventions in neonatal HIE. Puchowicz et al. demonstrated that diet-induced ketosis preserved mitochondrial respiration, reduced oxidative damage, and improved tissue survival following focal cerebral ischemia [91]. Similarly, Prins and colleagues reported age-dependent neuroprotection by ketones, with immature animals deriving significantly greater benefit than adults, underscoring the unique responsiveness of the developing brain to ketone-based metabolic support [92]. Although these models do not fully recapitulate neonatal HIE, they provide convergent evidence that ketones preferentially enhance mitochondrial efficiency and energy stability during early life.

Mechanistically, preclinical studies consistently indicate that ketone metabolism mitigates several downstream components of HIE injury. Ketogenic interventions reduce mitochondrial reactive oxygen species generation, improve redox balance, and enhance coupling efficiency, thereby limiting secondary energy failure [34,35]. These effects are particularly relevant in neonatal HIE, where oxidative stress and mitochondrial dysfunction drive delayed neuronal and oligodendroglial injury. In addition, transcriptomic and molecular studies suggest that KD modulate gene expression programs related to antioxidant defense, apoptosis, and synaptic stability in the developing brain, without overt evidence of impaired growth or gross neurodevelopment in animal models [93,94].

Despite these encouraging findings, some limitations of the current preclinical literature are present. Most studies, in fact, differ substantially in animal age, timing of ketogenic exposure, duration of ketosis, and outcome measures, and very few directly model the clinically relevant scenario of post-insult intervention or combination with TH. Moreover, data specifically addressing long-term neurodevelopmental outcomes after neonatal HIE under ketogenic conditions remain sparse. Nonetheless, taken together, available preclinical evidence supports the concept that ketogenic strategies—by stabilizing cerebral energy metabolism, reducing oxidative injury, and leveraging the unique metabolic physiology of the neonatal brain—represent a biologically compelling and developmentally tailored approach to neuroprotection in HIE.

### 4.10. Endogenous Ketosis and Dexamethasone Pretreatment

The study by Dardzinski et al. constitutes a landmark contribution to the understanding of ketone-mediated neuroprotection in HIE brain injury and remains one of the most mechanistically informative preclinical demonstrations of endogenous ketosis as a protective metabolic state. Using a postnatal day 7 (P7) rat model—developmentally comparable to the term human neonate—the authors showed that systemic pretreatment with dexamethasone induced a robust increase in circulating β-OHB prior to HIE, accompanied by a profound attenuation of subsequent cerebral injury [53].

From a mechanistic standpoint, the central insight of this work lies in the preservation of cerebral bioenergetics during and after the hypoxic–ischemic insult. In vivo ^31P magnetic resonance spectroscopy (^31P-MRS) revealed that dexamethasone-pretreated pups maintained near-normal levels of ATP and phosphocreatine throughout the insult and reperfusion phases, whereas untreated controls exhibited rapid depletion of high-energy phosphates, consistent with catastrophic failure of oxidative phosphorylation. This metabolic preservation closely paralleled histopathological outcomes: pretreated animals displayed minimal cortical and subcortical injury, while controls developed extensive neuronal loss and infarction [53].

Importantly, the authors systematically examined potential confounders and concluded that the observed neuroprotection was unlikely to be explained by improved oxygen delivery, altered cerebral blood flow, or nonspecific hemodynamic effects. Instead, dexamethasone pretreatment induced a marked metabolic shift characterized by reduced cerebral glucose utilization and increased reliance on ketone bodies as oxidative substrates. Given that ketone metabolism enters the tricarboxylic acid cycle downstream of glycolysis and bypasses several vulnerable steps of glucose-dependent energy production, this shift likely conferred a critical energetic advantage under conditions of limited oxygen and impaired mitochondrial function. In the context of neonatal HIE—where inhibition of pyruvate dehydrogenase, mitochondrial complex I dysfunction, and secondary energy failure are prominent—enhanced ketone availability may therefore stabilize ATP generation when glucose oxidation becomes inefficient or fails entirely [53].

At the cellular level, ketone-supported mitochondrial respiration is associated with a higher ATP/O_2_ ratio and reduced electron leak compared with glucose-based metabolism, thereby limiting the production of ROS during reperfusion. Although oxidative stress markers were not directly assessed in this study, the preservation of phosphocreatine and ATP strongly suggests improved mitochondrial coupling efficiency and resistance to energy collapse. These findings are particularly relevant to the immature brain, which exhibits both high energy demand and a limited capacity to upregulate alternative metabolic pathways in response to acute injury.

It is essential to acknowledge that dexamethasone exerts pleiotropic systemic effects, including modulation of stress hormone signaling and peripheral metabolism. However, the tight temporal and quantitative correlation between elevated plasma β-OHB levels, preserved cerebral high-energy phosphates, and dramatically reduced histological injury supports a causal role for endogenous ketosis in mediating neuroprotection.

From a translational perspective, the work by Dardzinski et al. has enduring relevance. It establishes that metabolic preconditioning via ketosis is feasible in the neonatal brain and can profoundly alter injury severity. At the same time, it highlights critical challenges for clinical translation, including the impracticality and safety concerns of steroid pretreatment and the narrow therapeutic window inherent to pre-injury interventions. These limitations have shifted subsequent research toward nutritional and exogenous ketone strategies capable of achieving similar metabolic effects without the systemic liabilities of glucocorticoids.

### 4.11. Differential Metabolism of Glucose Versus β-OHB in Neonatal Hypoxia–Ischemia

A growing body of experimental evidence indicates that the neonatal brain is metabolically distinct from the adult brain, exhibiting a developmental preference for ketone bodies over glucose as oxidative substrates. During late gestation and early postnatal life, ketone bodies contribute substantially to cerebral energy production and lipid biosynthesis, supporting rapid brain growth, myelination, and synaptogenesis [87,88]. This physiological reliance on ketone metabolism provides an essential framework for understanding why ketone availability may confer a selective advantage under pathological conditions such as HIE injury.

The most direct experimental demonstration of substrate-specific metabolic resilience in neonatal HIE was provided by Odorcyk et al., who quantitatively compared glucose and β-OHB metabolism using ^13C-labeled substrates and nuclear magnetic resonance spectroscopy in a rat model of neonatal HIE. Their data showed that, following HIE, oxidation of ^13C-glucose was markedly impaired, with reduced incorporation into TCA intermediates and increased lactate accumulation, reflecting a shift toward anaerobic glycolysis. In contrast, ^13C-β-OHB was efficiently incorporated into TCA metabolites, indicating preserved mitochondrial oxidation of ketone bodies despite the ischemic insult. This preferential utilization of β-OHB was accompanied by improved preservation of cerebral high-energy phosphates, strongly supporting the concept that ketone metabolism remains functional when glucose metabolism fails [95].

Mechanistically, these findings are highly relevant to the pathophysiology of neonatal HIE. Glucose-dependent ATP generation is particularly vulnerable to hypoxia–ischemia due to inhibition of pyruvate dehydrogenase, mitochondrial complex I dysfunction, and accumulation of lactate and protons during reperfusion. Ketone bodies, by entering the TCA cycle as acetyl-CoA independently of glycolysis and pyruvate dehydrogenase, bypass key metabolic bottlenecks and support oxidative phosphorylation under conditions of limited oxygen availability. The work of Odorcyk et al. therefore provides direct metabolic evidence that the immature brain possesses an intrinsic capacity to preferentially oxidize ketones during HIE, conferring a form of endogenous metabolic neuroprotection.

In addition, experimental models of cerebral ischemia outside the neonatal period have shown that ketone supplementation can enhance mitochondrial efficiency, reduce oxidative stress, and improve energy metabolism, suggesting that the bioenergetic benefits of ketones under ischemic conditions are conserved across developmental stages, albeit most pronounced in the immature brain [91]. Although these studies do not directly model neonatal HIE, they reinforce the mechanistic plausibility of ketone-based metabolic rescue.

More recent experimental work in neonatal HIE models has begun to explore the therapeutic implications of these metabolic differences. In post-insult paradigms, exogenous β-OHB administration has been associated with improved cerebral metabolic profiles and reduced neuronal injury, further supporting the idea that ketone availability during the phase of secondary energy failure may be particularly beneficial [96].

Taken together, these observations support a unifying model in which neonatal HIE unmasks a substrate-specific vulnerability of glucose metabolism, while ketone oxidation remains comparatively preserved. Exploiting this intrinsic metabolic asymmetry—through KDs or exogenous ketone supplementation—may therefore represent a rational strategy to stabilize cerebral bioenergetics during secondary energy failure in neonatal HIE.

### 4.12. Exogenous β-OHB and Post-Insult Neuroprotection

The possibility that ketone bodies may exert neuroprotective effects even when administered after HIE injury is of particular translational relevance for neonatal HIE. In this context, Lee et al. provided one of the first direct experimental demonstrations that exogenous β-OHB delivered post-insult can attenuate brain injury in the immature brain. Using a suckling rat model, the authors administered β-OHB following HIE and observed significant reductions in neuronal loss in vulnerable brain regions, accompanied by a decrease in TUNEL-positive cells, indicating attenuation of apoptotic cell death pathways [97]. These histopathological benefits were paralleled by improvements in functional outcomes, supporting the biological relevance of the observed tissue protection.

From a mechanistic perspective, although Lee et al. did not directly measure cerebral metabolic fluxes, the pattern of injury attenuation is consistent with stabilization of post-ischemic energy metabolism. The post-insult period in neonatal HIE is characterized by secondary energy failure, during which glucose utilization remains impaired due to mitochondrial dysfunction and inhibition of key glycolytic–oxidative coupling steps. Exogenous β-OHB, by serving as an efficient oxidative substrate that enters the TCA cycle independently of glycolysis and pyruvate dehydrogenase, may partially restore ATP generation during this vulnerable phase, thereby limiting the activation of delayed cell death cascades. The reduction in apoptotic markers observed in β-OHB-treated animals is compatible with improved mitochondrial function and energy availability during reperfusion [97].

Additional support for the therapeutic relevance of post-insult β-OHB administration comes from more recent neonatal HIE studies exploring metabolic and combinatorial strategies. In a neonatal HIE model, Fabres and colleagues demonstrated that exogenous β-OHB administered after injury increased cerebral glucose uptake and reduced hippocampal neuronal loss, with additive neuroprotective effects when combined with therapeutic hypothermia, the current standard of care for neonatal HIE [96]. These findings collectively suggest that ketone administration can favorably modulate post-ischemic cerebral metabolism and may synergize with hypothermia-based neuroprotection.

Moreover, experimental models of severe metabolic stress, such as hypoglycemia-induced neuronal injury, further reinforce the concept that β-OHB can preserve neuronal viability under conditions of impaired glucose availability. In such models, systemic β-OHB administration reduced neuronal death, preserved cellular energy levels, and attenuated ROS production, underscoring the capacity of ketone bodies to stabilize bioenergetics and limit oxidative damage when glucose metabolism is compromised [98].

Taken together, these studies indicate that exogenous β-OHB is not merely a preconditioning agent but can function as a post-insult metabolic therapy capable of attenuating evolving brain injury in the immature brain. While important questions remain regarding optimal dosing, timing, and interaction with established therapies such as TH, the convergence of evidence from neonatal HIE models and related metabolic injury paradigms provides a strong rationale for further translational exploration of ketone-based strategies in neonatal HIE.

### 4.13. Broader Ischemic Models

Although direct evidence in neonatal HIE remains limited, a substantial body of data from broader ischemic models supports the concept that ketone-based metabolism exerts robust neuroprotective effects across different forms of cerebral ischemia. Zharikova et al. recently provided an integrative analysis of transcriptomic, metabolomic, and microbiome changes induced by KD in experimental stroke models, collating evidence from adult and juvenile animals subjected to focal cerebral ischemia. Across these models, KD consistently reduced infarct volumes, improved neurological and behavioral outcomes, and attenuated molecular markers of apoptosis, oxidative stress, and neuroinflammation [99]. Importantly, the authors highlighted coordinated metabolic reprogramming toward enhanced mitochondrial function and antioxidant capacity as central mediators of these effects.

These findings are consistent with earlier preclinical studies demonstrating that ketogenic interventions or exogenous ketone administration confer protection in adult models of ischemic stroke. Puchowicz et al. showed that diet-induced ketosis preserved mitochondrial respiration and reduced oxidative damage following focal ischemia, with improved tissue survival and energy metabolism [91]. Similarly, Suzuki and colleagues demonstrated that ketone administration after transient cerebral ischemia reduced infarct size and improved neurological recovery, effects linked to improved mitochondrial redox state and reduced ROS generation [100]. While these studies were conducted in mature brains, they provide strong mechanistic support for the idea that ketone metabolism enhances resilience to ischemic energy failure.

From a mechanistic standpoint, these broader ischemic models converge on several pathways directly relevant to neonatal HIE. Ketone metabolism improves the ATP/O_2_ ratio, limits mitochondrial electron leak, and reduces oxidative stress during reperfusion—processes that are central drivers of delayed injury in both adult stroke and neonatal HIE. Transcriptomic analyses further suggest that KD modulates gene expression programs related to mitochondrial biogenesis, antioxidant defenses, and inflammatory signaling, including suppression of pro-apoptotic and pro-inflammatory pathways [93,99]. Although the specific gene networks engaged may differ by developmental stage, the directionality of these effects aligns closely with the pathophysiology of neonatal HIE.

Critically, extrapolation from adult ischemic models to neonatal HIE must be performed cautiously.

However, evidence from adult and juvenile ischemic models strengthens the biological plausibility of ketone-mediated neuroprotection and provides important mechanistic insights that complement neonatal-specific studies. While these data cannot substitute for direct neonatal HIE experiments, they reinforce the concept that targeting cerebral metabolism through ketogenic or ketone-based strategies addresses conserved mechanisms of ischemic injury—mitochondrial dysfunction, oxidative stress, and programmed cell death—that are shared across age groups. In this framework, neonatal HIE emerges not as an exception, but as a developmentally primed condition in which the neuroprotective potential of ketone metabolism may be particularly pronounced. Given the high degree of interconnection among metabolic, inflammatory, and developmental mechanisms, partial overlap across the following sections is unavoidable.

## 5. Clinical Evidence: What Can We Realistically Look Inside Today?

### 5.1. Ketogenic Diet in Infants and Neonates

Over the past decade, a growing body of evidence has established that the KD is feasible, safe, and often effective in infants younger than 2 years, including neonates with severe and refractory epilepsies [101]. Multiple cohort studies and systematic reviews have demonstrated that seizure reduction rates in infants are comparable to those observed in older children, provided that the diet is implemented within specialized centers with careful nutritional and metabolic monitoring [102,103,104]. Importantly, these studies have challenged earlier assumptions that infants are unable to tolerate or sustain ketosis, instead showing that early life represents a physiologically ketone-adapted state.

In a recent multicenter feasibility study, Hsieh et al. evaluated different KD variants—including classic KD, MCT-KD, and modified KD—in infants with drug-resistant epilepsy and reported that all approaches were implementable with acceptable safety profiles when rigorous protocols for glucose, acid–base balance, lipid levels, and growth parameters were applied [105]. Although transient adverse effects such as hypoglycemia, metabolic acidosis, dyslipidemia, and gastrointestinal intolerance were observed, these complications were generally manageable and rarely required discontinuation of therapy. Similar findings have been reported in earlier infant cohorts, reinforcing that age alone should not be considered a contraindication to KD [101]. Experience specifically in neonates is necessarily more limited, but emerging case series and small observational studies are particularly informative. Neonatal KD has been successfully employed in refractory seizures due to diverse etiologies, including genetic epileptic encephalopathies, metabolic disorders, and acute symptomatic seizures, with reports of meaningful seizure reduction and acceptable tolerability [106,107,108]. Notably, KD has been administered even in premature or medically fragile neonates under intensive monitoring, demonstrating that ketosis can be achieved and maintained in this population without prohibitive risk when managed by experienced multidisciplinary teams [107]. From a mechanistic perspective, the apparent tolerability of KD in neonates aligns with developmental neurobiology. During late gestation and early postnatal life, ketone bodies contribute substantially to cerebral energy metabolism and lipid synthesis, supporting myelination and membrane formation. Neonates exhibit high expression of monocarboxylate transporters and ketolytic enzymes, rendering the neonatal brain particularly efficient at ketone uptake and utilization. This physiological predisposition likely explains why infants and neonates can achieve stable ketosis at lower ketogenic ratios than older children, potentially reducing metabolic stress while preserving efficacy. These clinical experiences in neonatal epilepsy are highly relevant for HIE. They demonstrate that KD-based interventions are technically feasible even in critically ill neonates and can be delivered safely in intensive care settings when appropriate expertise is available. While seizure control is the primary endpoint in epilepsy cohorts, the observed safety and metabolic adaptability provide an essential translational foundation for exploring KD or ketone-based strategies in neonatal HIE, where metabolic failure, excitotoxicity, and network instability are central drivers of injury. Importantly, these data suggest that the neonatal period—often perceived as too fragile for metabolic interventions—may in fact represent a uniquely permissive window for ketone-based neuroprotection.

### 5.2. KD in Acquired Structural Etiologies Including HIE

Although most pediatric studies of KD focus on genetic and developmental epileptic encephalopathies, accumulating evidence suggests that KD can also be effective in seizures arising from acquired structural brain injuries, including hypoxic–ischemic injury. In a retrospective cohort of children with refractory epilepsy due to acquired structural etiologies, Villaluz et al. found that a majority of patients responded favorably to KD, with a significant proportion demonstrating >50% reduction in seizure frequency at 3 months and sustained improvement over longer follow-up (7/9 responders at 3 months in the acquired group) [109]. Importantly, among this cohort were patients with documented HIE, of whom two achieved seizure freedom at 6 months, suggesting that KD may favorably modulate epileptogenesis even when the underlying lesion is structural and non-genetic.

This pattern of efficacy is supported by larger retrospective analyses. In a single-center study of 42 children with drug-resistant epilepsy due to structural pathology—including neonatal HIE, hypoglycemic injury, intracranial hemorrhage, and sequelae of infection—KD therapy was associated with meaningful seizure control and functional improvements over 1–6 months of treatment (seizure control effective rate ~52.4% at 3 months), although response rates varied across structural subgroups (including HIE) [110].

Similarly, a retrospective analysis of 23 children treated with KD for structural etiologies reported a high proportion of responders (60.9% at 3 months), with particularly robust seizure reductions seen in the subgroup of patients with a history of neonatal brain injury, including HIE (100% > 50% reduction in seizures in the HIE subgroup) [111].

These findings align with the broader literature indicating that KD can be effective across a range of structural causes of drug-resistant epilepsy, and that certain acquired injuries—including those of perinatal onset—may be particularly responsive.

Beyond purely seizure frequency outcomes, clinical series also note improvements in cognitive, behavioral, and developmental domains in children on KD following structural brain injury. In the larger cohort described by Zhang et al., up to 69% of patients exhibited cognitive and behavioral benefits coinciding with improved seizure control, underscoring the potential of KD to influence neurodevelopmental trajectories in structurally injured brains [110].

Mechanistically, several pathways may contribute to KD’s efficacy in structural epilepsies. Ketone bodies exert anticonvulsant effects through modulation of excitatory transmission, enhancement of inhibitory tone, stabilization of neuronal membrane potential, and reduction in neuroinflammation and oxidative stress—mechanisms that are relevant irrespective of the initial lesion etiology. Moreover, structural injuries such as HIE often leave a milieu of metabolic dysfunction, network reorganization, and gliosis that may be particularly amenable to metabolic modulation via KD. Although direct mechanistic studies in acquired structural epilepsy are sparse, the consistency of clinical responses across etiologies suggests that KD’s effects extend beyond specific genetic substrates and tap into more general neuroprotective and neuromodulatory pathways.

### 5.3. Specific Evidence Synthesis for KD in Neonatal HIE

The review by Zhou et al. represents, to date, the most comprehensive and focused synthesis addressing the potential role of KD and ketone-based strategies in neonatal HIE [9]. By integrating developmental neurobiology, experimental hypoxia–ischemia models, and early clinical observations, the authors articulate a coherent framework in which ketone metabolism emerges as a biologically plausible and developmentally aligned neuroprotective intervention for the injured newborn brain.

Zhou and colleagues emphasize that the mechanistic rationale for KD in neonatal HIE is both strong and intrinsically multi-layered. Ketone bodies target central nodes of hypoxic–ischemic injury, including mitochondrial bioenergetic failure, excitotoxic glutamatergic signaling, oxidative stress, neuroinflammation, and epigenetic dysregulation. Rather than acting on a single pathway, ketones operate as pleiotropic neurometabolic modulators, capable of stabilizing energy metabolism, dampening inflammatory cascades, and reshaping transcriptional programs critical for cell survival and network maturation. This systems-level mode of action is particularly relevant in HIE, where injury evolves across tightly interwoven metabolic, inflammatory, and developmental axes [9].

From a preclinical standpoint, animal models of neonatal hypoxia–ischemia consistently demonstrate reduced brain injury and improved functional outcomes when ketone availability is increased, either through dietary manipulation, pharmacological induction of ketosis, or exogenous ketone administration. Importantly, protective effects have been observed both when ketosis precedes the insult—buffering the initial energetic collapse—and when ketones are supplied after hypoxia–ischemia, attenuating secondary energy failure, oxidative stress, and delayed cell death [9]. These findings resonate with broader ischemia literature and reinforce the notion that ketone metabolism can intervene at multiple temporal stages of injury evolution.

On the clinical side, Zhou et al. contextualize neonatal HIE within the expanding experience of KD in neonates and infants with refractory epilepsy. They underscore that KD is feasible and often effective even in very young patients, including those with seizures secondary to hypoxic–ischemic brain injury, provided that implementation occurs in specialized settings with meticulous metabolic and nutritional monitoring [9]. While seizure control is not synonymous with neuroprotection, these data are crucial in establishing the safety, tolerability, and practical deliverability of ketogenic strategies in precisely the population most relevant to HIE.

Despite strong mechanistic plausibility and converging preclinical evidence, no interventional trials have yet tested whether ketone-based metabolic support can enhance neuroprotection beyond hypothermia alone in neonatal HIE. This gap persists despite the well-recognized limitations of hypothermia, which leaves a substantial proportion of infants with death or long-term neurodisability.

## 6. Discussion

Our scoping review synthesizes a growing body of mechanistic, preclinical, and emerging clinical evidence suggesting that ketogenic strategies may represent a biologically plausible adjunctive approach in neonatal HIE. Unlike conventional neuroprotective paradigms that primarily target single downstream injury pathways, ketone-based metabolism exerts pleiotropic effects across multiple levels of the ischemic cascade, including mitochondrial energetics, excitotoxic signaling, neuroinflammation, epigenetic regulation, and white matter integrity [6,9,10,12].

A central insight emerging from experimental studies is the intrinsic metabolic advantage of the immature brain when ketone bodies are available. Neonatal neurons and glia readily oxidize β-OHB and AcAC, which enter the TCA cycle downstream of complex I, a major site of hypoxia–ischemia-induced dysfunction. This bypass confers greater energetic efficiency, reduces reactive oxygen species generation, and preserves high-energy phosphate stores during secondary energy failure [53,95]. Importantly, both endogenous ketoses induced by dexamethasone pretreatment and exogenous post-insult β-OHB administration have demonstrated neuroprotective effects in neonatal rodent models, supporting the concept that ketone availability before or after injury can mitigate neuronal loss [53,97]. Beyond energy metabolism, ketone bodies modulate excitatory–inhibitory balance through mechanisms particularly relevant to the neonatal brain. Decanoic acid, a key component of the medium-chain triglyceride KD, acts as a direct non-competitive antagonist of AMPA receptors, thereby attenuating glutamate-driven excitotoxicity and epileptiform propagation independent of GABAergic pathways [62,64]. This feature is especially pertinent in early development, when depolarizing GABA signaling limits the efficacy of conventional antiseizure medications.

Neuroinflammation represents another critical target of ketone-mediated neuroprotection. Β-OHB has been shown to inhibit the NLRP3 inflammasome, reduce microglial activation, and suppress pro-inflammatory cytokine release in multiple experimental systems [4,36]. Although direct evidence in neonatal HIE models remains limited, the prolonged inflammatory response characteristic of neonatal brain injury renders this mechanism highly relevant, as emphasized in recent integrative reviews [9,12].

Epigenetic regulation further distinguishes ketone bodies from traditional metabolic substrates. Acting as endogenous histone deacetylase inhibitors, ketones influence transcriptional programs related to oxidative stress resistance, neuronal survival, and synaptic plasticity [10]. Given the heightened epigenetic plasticity of the perinatal brain, such effects may extend neuroprotection beyond the acute phase, potentially shaping long-term neurodevelopmental trajectories after HIE. The evidences are summarized in Table 1.

**Figure 2:**
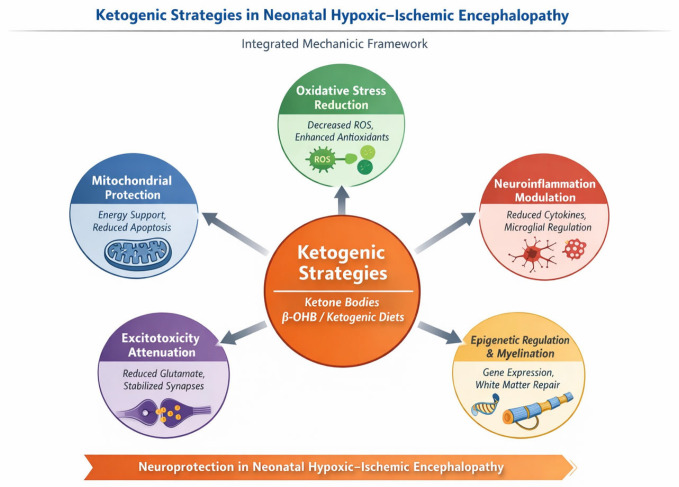
Integrated mechanistic framework of ketogenic strategies in neonatal hypoxic–ischemic encephalopathy (HIE). The figure summarizes the proposed multimodal mechanisms through which ketogenic strategies and ketone bodies may contribute to neuroprotection in the immature brain following hypoxic–ischemic injury. Ketogenic interventions are depicted as central metabolic modulators influencing interconnected pathways, including mitochondrial bioenergetics, oxidative stress, excitotoxicity, neuroinflammation, and longer-term epigenetic and white matter processes. Arrows indicate functional interactions rather than direct causal relationships, highlighting the pleiotropic and hypothesis-generating nature of this framework.

Given the multifactorial nature of neonatal hypoxic–ischemic brain injury, Figure 2 schematically summarizes the integrated mechanistic framework through which ketogenic strategies and ketone bodies may exert pleiotropic neuroprotective effects. By targeting convergent metabolic, inflammatory, and developmental pathways, ketogenic interventions are conceptually positioned as multimodal adjunctive strategies rather than single-mechanism therapies.

From a clinical standpoint, accumulating evidence indicates that KDs can be safely implemented in infants, including very young and medically complex patients, when managed in specialized centers. Multiple cohort studies and systematic analyses demonstrate feasibility, acceptable tolerability, and meaningful seizure reduction in infants with refractory epilepsies, including those with acquired structural etiologies such as hypoxic–ischemic injury [109,110]. These observations, although not neuroprotective trials per se, provide important proof-of-principle that ketone-based therapies are clinically viable in populations relevant to HIE survivors. An important unresolved issue concerns which ketogenic approach may be most appropriate in the context of neonatal HIE. Rather than representing a single intervention, “ketogenic strategies” encompass a spectrum of metabolic tools, including classic ketogenic diets, medium-chain triglyceride-based diets, exogenous ketone supplementation (e.g., β-OHB), and targeted fatty acids such as decanoic acid. Each of these approaches may differ substantially in terms of feasibility, onset of action, and mechanistic emphasis.

From a clinical perspective, several practical issues must be considered before ketogenic strategies can be translated into neonatal HIE care. These include safety and tolerability in critically ill neonates, metabolic monitoring during therapeutic hypothermia, potential interactions with glucose and lipid metabolism, and the feasibility of dietary versus exogenous ketone approaches.

Exogenous ketone supplementation may offer theoretical advantages in the acute phase by providing rapidly available alternative substrates without the need for major dietary modifications. Conversely, dietary ketogenic strategies may be more suitable for later phases, where sustained metabolic modulation could influence neuroinflammation and recovery. At present, however, these considerations remain theoretical and require rigorous clinical evaluation.

Conceptually, different strategies may align with distinct phases of the triphasic evolution of HIE. During the acute and early secondary energy failure phases, rapidly bioavailable substrates such as exogenous ketone bodies may be better suited to support cerebral bioenergetics when glucose oxidation is impaired. In contrast, dietary ketogenic approaches may be more relevant in the subacute or tertiary phases, where sustained modulation of inflammation, network excitability, and metabolic programming may influence longer-term recovery trajectories.

At present, however, optimal timing, dosing, duration, and patient selection remain undefined, and no comparative data exist to support one ketogenic strategy over another in neonatal HIE. These variables represent critical priorities for future preclinical and clinical studies.

Finally, recent studies on nutritional management during TH challenge long-standing assumptions regarding feeding intolerance and metabolic vulnerability in HIE. Carefully monitored enteral and parenteral nutrition during hypothermia appears feasible and safe, opening a translational window for metabolically targeted interventions during the acute phase of injury [14,16,17,18,90]. Together, these data suggest that metabolic modulation in HIE is not only biologically justified but also clinically actionable. Clinical and preclinical evidences are reported in Table 2.

Despite these advances, major knowledge gaps remain. No randomized controlled trials have evaluated ketogenic diets or exogenous ketones as adjuncts to therapeutic hypothermia, and optimal timing, dosing, formulation, and patient selection remain undefined [9]. Addressing these gaps will require rigorously designed translational studies that integrate developmental neurobiology, metabolism, and ethical considerations unique to neonatal care. Importantly, we acknowledge that the present scoping review is hypothesis-generating rather than practice-changing. To date, no randomized controlled trials or prospective interventional studies have directly evaluated ketogenic diets or exogenous ketone supplementation in neonates with hypoxic–ischemic encephalopathy. Therefore, the clinical implications discussed herein are necessarily extrapolated from developmental neurobiology, neonatal and juvenile preclinical models, and clinical experience with ketogenic therapies in infants with refractory epilepsy and acquired brain injury.

This scoping review has several limitations. First, the majority of available evidence derives from preclinical models, with limited direct clinical data in neonates with HIE. Second, significant heterogeneity exists in experimental paradigms, timing of intervention, and outcome measures, limiting cross-study comparability. Third, the absence of randomized controlled trials precludes conclusions regarding efficacy, safety, and optimal implementation of ketogenic strategies in this population.

Future studies should prioritize phase-specific interventions aligned with the temporal evolution of HIE, comparative evaluation of different ketogenic approaches, and the integration of metabolic biomarkers with neurodevelopmental outcomes. Moreover, the absence of randomized clinical trials in neonates with HIE precludes any conclusions regarding efficacy, safety, or optimal implementation of ketogenic strategies in this population. These limitations underscore the need for rigorously designed translational studies integrating metabolic, neurodevelopmental, and ethical considerations unique to neonatal care.

## 7. Conclusions

Ketone-based metabolic strategies represent a compelling and underexplored avenue for neuroprotection in neonatal HIE. By simultaneously targeting energy failure, excitotoxicity, inflammation, epigenetic programming, and white matter vulnerability, ketone bodies align closely with the multifactorial nature of neonatal brain injury. Robust preclinical evidence, combined with growing clinical experience in infants, supports the feasibility of translating these approaches into carefully designed clinical trials. At present, the available evidence does not support the use of ketogenic interventions as standard clinical practice in neonatal HIE. Rather, the existing data define a strong biological and developmental rationale that warrants further translational investigation and carefully designed clinical trials. Future studies should focus on defining optimal therapeutic windows, delivery strategies, and outcome measures, with the ultimate goal of complementing therapeutic hypothermia and improving long-term neurodevelopmental outcomes in this vulnerable population.

## Figures and Tables

**Table 1:** Ketone-mediated mechanisms relevant to neuroprotection in neonatal hypoxic–ischemic encephalopathy.

Mechanistic Domain	Key Targets	Relevance in HIE	Supporting Evidence
Mitochondrial energetics	β-OHB enter TCA cycle as acetyl-CoA; improved ATP/O_2_ ratio; preservation of mitochondrial membrane potential	Secondary energy failure and complex I dysfunction are central to neonatal HIE injury	Dardzinski et al. [53]; Odorcyk et al. [32]; Jang et al. [9]
Oxidative stress	Reduced ROS generation; reduced lipid peroxidation	Oxidative injury and oligodendrocyte vulnerability drive white matter damage	Makievskaya et al. [6]; Brandt et al. [21]
Excitotoxicity	AMPA receptor inhibition by decanoic acid; reduced glutamate release; enhanced astrocytic uptake	AMPA-mediated excitation propagates seizures; GABA is depolarizing in neonates	Chang et al. [62]; Rogawski et al. [64]
Neuroinflammation	Inhibition of NLRP3 inflammasome; reduced IL-1β, TNF-α, IL-6; modulation of microglial phenotype	Neuroinflammation is prolonged and contributes to secondary injury in HIE	Youm et al. [36], Qi et al. [4]; Wood et al. [12]
Epigenetic regulation	Class I/IIa HDAC inhibition; transcription of antioxidant and plasticity-related genes	Perinatal brain is highly epigenetically plastic; long-term programming effects	Jang et al. [10]
White matter	Support of lipid synthesis, myelination, oligodendrocyte maturation	Diffuse white matter injury underlies later motor and cognitive deficits	Jang et al. [10]; Makievskaya et al. [6]

β-OHB: β-hydroxybutyrate; TCA: Tricarboxylic Acid Cycle; ATP: Adenosine Triphosphate; HIE: Hypoxic–ischemic encephalopathy; ROS: Reactive oxygen species; AMPA: α-amino-3-hydroxy-5-methyl-4-isoxazolepropionic acid; GABA: Gamma-aminobutyric acid; NLRP3: NOD-like receptor family pyrin domain containing 3; IL-1β: Interleukin-1β; TNF-α: Tumor necrosis factor-α; IL-6: Interleukin-6; HDAC: Class I histone deacetylase.

**Table 2:** Experimental and clinical evidence supporting ketogenic strategies in neonatal hypoxic–ischemic brain injury.

Study/Model	Intervention	Timing Relative to HI	Main Outcomes
Dardzinski et al. [53], P7 rat	Dexamethasone-induced endogenous ketosis	Pre-insult	Preserved ATP and phosphocreatine; minimal cortical injury
Odorcyk et al. [95], neonatal rat	Exogenous β-OHB vs. glucose (^13^C tracing)	During and post-HIE	Superior β-OHB oxidation; reduced lactate accumulation
Lee et al. [97], P13 rat	Exogenous β-OHB	Post-insult	Reduced neuronal loss and apoptosis; improved function
Chang et al. [62], hippocampal slices	Decanoic acid (MCT-KD component)	Acute	Non-GABAergic antiseizure mechanism
Zhou et al. [9], integrative review	KD and ketone bodies	Pre- and post-HIE	Framework for neonatal HIE translation

ATP: Adenosine Triphosphate; β-OHB: β-hydroxybutyrate; HIE: Hypoxic–ischemic encephalopathy; MCT: Medium-chain triglyceride; KD: Ketogenic Diet; GABA: Gamma-aminobutyric acid.

## Data Availability

No new data were created or analyzed in this study. Data sharing is not applicable to this article.

## Abbreviations

The following abbreviations are used in this manuscript:

HIE	Hypoxic–ischemic encephalopathy
TH	Therapeutic hypothermia
KD	Ketogenic diet
AcAc	Acetoacetate
β-OHB	β-hydroxybutyrate
ATP	Adenosine Triphosphate
NMDA	N-methyl-D-aspartate
AMPA	α-amino-3-hydroxy-5-methyl-4-isoxazolepropionic acid
P-MRS	Phosphorus magnetic resonance spectroscopy
ROS	Reactive oxygen species
MRI	Magnetic Resonance Imaging
MCT1	Monocarboxylate Transporter 1
MCT2	Monocarboxylate Transporter 2
SCOT	Succinyl-CoA:3-oxoacid CoA transferase
TCA	Tricarboxylic Acid Cycle
NLRP3	NOD-like receptor family pyrin domain containing 3
IL-1β	Interleukin-1β
IL-18	Interleukin-18
TNF-α	Tumor necrosis factor-α
IL-6	Interleukin-6
GABA	Gamma-aminobutyric acid
HDACs	Class I histone deacetylases
FOXO3A	Forkhead box O3A
Nrf2	Nuclear factor erythroid 2–related factor 2
NKCC1	Sodium-Potassium-Chloride Cotransporter 1
KCC2	Potassium Chloride Cotransporter 2
MCT	Medium-chain triglyceride
GluA2	A subunit of AMPA receptor
TUNEL	Terminal deoxynucleotidyl transferase dUTP nick-end labeling

## References

[1] Jacobs S.E., Berg M., Hunt R., Tarnow-Mordi W.O., Inder T.E., Davis P.G. (2013). Cooling for newborns with hypoxic ischaemic encephalopathy. Cochrane Database Syst. Rev..

[2] Korf J.M., McCullough L.D., Caretti V. (2023). A narrative review on treatment strategies for neonatal hypoxic ischemic encephalopathy. Transl. Pediatr..

[3] Chan N.H., Hawkins C.C., Rodrigues B.V., Cornet M.C., Gonzalez F.F., Wu Y.W. (2025). Neuroprotection for neonatal hypoxic-ischemic encephalopathy: A review of novel therapies evaluated in clinical studies. Dev. Med. Child Neurol..

[4] Qin X., Cheng J., Zhong Y., Mahgoub O.K., Akter F., Fan Y., Aldughaim M., Xie Q., Qin L., Gu L. (2019). Mechanism and Treatment Related to Oxidative Stress in Neonatal Hypoxic-Ischemic Encephalopathy. Front. Mol. Neurosci..

[5] Armour E.A., Curcio A.M., Fryer R.H. (2020). Neonatal Hypoxic Ischemic Encephalopathy: An Updated Preclinical and Clinical Review. OBM Neurobiol..

[6] Makievskaya C.I., Popkov V.A., Andrianova N.V., Liao X., Zorov D.B., Plotnikov E.Y. (2023). Ketogenic Diet and Ketone Bodies against Ischemic Injury: Targets, Mechanisms, and Therapeutic Potential. Int. J. Mol. Sci..

[7] Pizzo F., Collotta A.D., Di Nora A., Costanza G., Ruggieri M., Falsaperla R. (2022). Ketogenic diet in pediatric seizures: A randomized controlled trial review and meta-analysis. Expert Rev. Neurother..

[8] Liu J., E R.H., Zhang J.Q., Wang D.P. (2025). Impact of a high-fat, low-carbohydrate ketogenic diet on seizure frequency in children with drug-resistant epilepsy: A systematic review and Meta-analysis. Front. Nutr..

[9] Zhou Y., Sun L., Wang H. (2023). Ketogenic Diet for Neonatal Hypoxic-Ischemic Encephalopathy. ACS Chem. Neurosci..

[10] Jang J., Kim S.R., Lee J.E., Lee S., Son H.J., Choe W., Yoon K.S., Kim S.S., Yeo E.J., Kang I. (2023). Molecular Mechanisms of Neuroprotection by Ketone Bodies and Ketogenic Diet in Cerebral Ischemia and Neurodegenerative Diseases. Int. J. Mol. Sci..

[11] Plourde G., Roumes H., Suissa L., Hirt L., Doche É., Pellerin L., Bouzier-Sore A.K., Quintard H. (2024). Neuroprotective effects of lactate and ketone bodies in acute brain injury. J. Cereb. Blood Flow Metab..

[12] Wood T.R., Stubbs B.J., Juul S.E. (2018). Exogenous Ketone Bodies as Promising Neuroprotective Agents for Developmental Brain Injury. Dev. Neurosci..

[13] Kosiek W., Rauk Z., Szulc P., Cichy A., Rugieł M., Chwiej J., Janeczko K., Setkowicz Z. (2022). Ketogenic diet impairs neurological development of neonatal rats and affects biochemical composition of maternal brains: Evidence of functional recovery in pups. Brain Struct. Funct..

[14] Gale C., Longford N.T., Jeyakumaran D., Ougham K., Battersby C., Ojha S., Dorling J. (2021). Feeding during neonatal therapeutic hypothermia, assessed using routinely collected National Neonatal Research Database data: A retrospective, UK population-based cohort study. Lancet Child Adolesc. Health.

[15] de Havilland A., Hariharan G. (2022). Is enteral feeding safe during therapeutic hypothermia in neonates with hypoxic–ischaemic encephalopathy?. Acta Paediatr..

[16] Molina Stornelli I., Bliznyuk N., Roig J.C., Neu J., Kisilewicz K., Rajderkar D., Sura L., Patel S., Chong D., Vadakal S. (2025). Nutrition in infants with hypoxic–ischemic encephalopathy: Insights from a single-center experience on parenteral and enteral feeding during therapeutic hypothermia. J. Matern. Fetal Neonatal Med..

[17] Samaai I., Pepper M.S., Pillay S., Horn A.R. (2025). Minimal impact of feed intolerance during therapeutic hypothermia for hypoxic ischaemic encephalopathy in a South African cohort with a standardised feeding regimen. Front. Pediatr..

[18] Martinovski H., Khanal L., Kraft D., Natarajan G. (2025). Enteral feeding in neonatal hypoxic–ischemic encephalopathy. Am. J. Perinatol..

[19] Hu Y., Chen F., Xiang X., Wang F., Hua Z., Wei H. (2022). Early versus delayed enteral nutrition for neonatal hypoxic–ischemic encephalopathy undergoing therapeutic hypothermia: A randomized controlled trial. Ital. J. Pediatr..

[20] Shui Z., Liu Y., Duan H., Sun Q., He H., He H., Wang J., Yin H. (2025). Predictive model for feeding intolerance in neonates with hypoxic ischemic encephalopathy during therapeutic hypothermia. Sci. Rep..

[21] Brandt M.J.V., Nijboer C.H., Nessel I., Mutshiya T.R., Michael-Titus A.T., Counotte D.S., Schipper L., van der Aa N.E., Benders M.J.N.L., de Theije C.G.M. (2021). Nutritional supplementation reduces lesion size and neuroinflammation in a sex-dependent manner in a mouse model of perinatal hypoxic–ischemic brain injury. Nutrients.

[22] Sanches E., van de Looij Y., Sow S., Toulotte A., da Silva A., Modernell L., Sizonenko S. (2021). Dose-Dependent Neuroprotective Effects of Bovine Lactoferrin Following Neonatal Hypoxia-Ischemia in the Immature Rat Brain. Nutrients.

[23] Greco P., Nencini G., Piva I., Scioscia M., Volta C.A., Spadaro S., Neri M., Bonaccorsi G., Greco F., Cocco I. (2020). Pathophysiology of hypoxic-ischemic encephalopathy: A review of the past and a view on the future. Acta Neurol. Belg.

[24] Yang M., Wang K., Liu B., Shen Y., Liu G. (2025). Hypoxic-Ischemic Encephalopathy: Pathogenesis and Promising Therapies. Mol. Neurobiol..

[25] Davidson J.O., Gonzalez F., Gressens P., Gunn A.J., Newborn Brain Society Guidelines and Publications Committee (2021). Update on mechanisms of the pathophysiology of neonatal encephalopathy. Semin. Fetal Neonatal Med..

[26] Vannucci R.C., Towfighi J., Vannucci S.J. (2004). Secondary energy failure after cerebral hypoxia-ischemia in the immature rat. J. Cereb. Blood Flow Metab..

[27] Gom R.C., Bhatt D., Villa B.R., George A.G., Lohman A.W., Mychasiuk R., Rho J.M., Teskey G.C. (2021). The ketogenic diet raises brain oxygen levels, attenuates postictal hypoxia, and protects against learning impairments. Neurobiol. Dis..

[28] Muneta T., Kawaguchi E., Nagai Y., Matsumoto M., Ebe K., Watanabe H., Bando H. (2016). Ketone body elevation in placenta, umbilical cord, newborn and mother in normal delivery. Glycative Stress Res..

[29] Felmlee M.A., Jones R.S., Rodriguez-Cruz V., Follman K.E., Morris M.E. (2020). Monocarboxylate Transporters (SLC16): Function, Regulation, and Role in Health and Disease. Pharmacol. Rev..

[30] Mikrogeorgiou A., Xu D., Ferriero D.M., Vannucci S.J. (2018). Assessing Cerebral Metabolism in the Immature Rodent: From Extracts to Real-Time Assessments. Dev. Neurosci..

[31] Westi E.W., Andersen J.V., Aldana B.I. (2023). Using stable isotope tracing to unravel the metabolic components of neurodegeneration: Focus on neuron-glia metabolic interactions. Neurobiol. Dis..

[32] Odorcyk F.K., Ribeiro R.T., Roginski A.C., Duran-Carabali L.E., Couto-Pereira N.S., Dalmaz C., Wajner M., Netto C.A. (2021). Differential Age-Dependent Mitochondrial Dysfunction, Oxidative Stress, and Apoptosis Induced by Neonatal Hypoxia-Ischemia in the Immature Rat Brain. Mol. Neurobiol..

[33] Gazerani P. (2025). The neuroplastic brain: Current breakthroughs and emerging frontiers. Brain Res..

[34] Sullivan P.G., Rippy N.A., Dorenbos K., Concepcion R.C., Agarwal A.K., Rho J.M. (2004). The ketogenic diet increases mitochondrial uncoupling protein levels and activity. Ann. Neurol..

[35] Kim D.Y., Davis L.M., Sullivan P.G., Maalouf M., Simeone T.A., van Brederode J., Rho J.M. (2007). Ketone bodies are protective against oxidative stress in neocortical neurons. J. Neurochem..

[36] Youm Y.H., Nguyen K.Y., Grant R.W., Goldberg E.L., Bodogai M., Kim D., D′Agostino D., Planavsky N., Lupfer C., Kanneganti T.D. (2015). The ketone metabolite β-hydroxybutyrate blocks NLRP3 inflammasome-mediated inflammatory disease. Nat. Med..

[37] Shimazu T., Hirschey M.D., Newman J., He W., Shirakawa K., Le Moan N., Grueter C.A., Lim H., Saunders L.R., Stevens R.D. (2013). Suppression of oxidative stress by β-hydroxybutyrate, an endogenous histone deacetylase inhibitor. Science.

[38] Hagberg H., Mallard C., Ferriero D.M., Vannucci S.J., Levison S.W., Vexler Z.S., Gressens P. (2015). The role of inflammation in perinatal brain injury. Nat. Rev. Neurol..

[39] Bi H., Le K., Xiong Q., Hu J., Wen H., Yu J., Liu Y., Yau S.Y., Song Z. (2025). Aucubin attenuates neonatal hypoxic-ischemic brain injury by suppressing NLRP3 inflammasome-mediated microglial pyroptosis. Bioorganic Chem..

[40] Sleiman S.F., Henry J., Al-Haddad R., El Hayek L., Abou Haidar E., Stringer T., Ulja D., Karuppagounder S.S., Holson E.B., Ratan R.R. (2016). Exercise promotes the expression of brain derived neurotrophic factor (BDNF) through the action of the ketone body β-hydroxybutyrate. Elife.

[41] Rubio C., López-Landa A., Romo-Parra H., Rubio-Osornio M. (2025). Impact of the Ketogenic Diet on Neurological Diseases: A Review. Life.

[42] García-Rodríguez D., Giménez-Cassina A. (2021). Ketone Bodies in the Brain Beyond Fuel Metabolism: From Excitability to Gene Expression and Cell Signaling. Front. Mol. Neurosci..

[43] He Y., Cheng X., Zhou T., Li D., Peng J., Xu Y., Huang W. (2023). β-Hydroxybutyrate as an epigenetic modifier: Underlying mechanisms and implications. Heliyon.

[44] Cornuti S., Chen S., Lupori L., Finamore F., Carli F., Samad M., Fenizia S., Caldarelli M., Damiani F., Raimondi F. (2023). Brain histone beta-hydroxybutyrylation couples metabolism with gene expression. Cell. Mol. Life Sci..

[45] Bustelo M., Barkhuizen M., van den Hove D.L.A., Steinbusch H.W.M., Bruno M.A., Loidl C.F., Gavilanes A.W.D. (2020). Clinical Implications of Epigenetic Dysregulation in Perinatal Hypoxic-Ischemic Brain Damage. Front. Neurol..

[46] Lodygensky G.A., Vasung L., Sizonenko S.V., Hüppi P.S. (2010). Neuroimaging of cortical development and brain connectivity in human newborns and animal models. J. Anat..

[47] Xie Z., Zhang D., Chung D., Tang Z., Huang H., Dai L., Qi S., Li J., Colak G., Chen Y. (2016). Metabolic Regulation of Gene Expression by Histone Lysine β-Hydroxybutyrylation. Mol. Cell..

[48] Yang H., Shan W., Zhu F., Wu J., Wang Q. (2019). Ketone Bodies in Neurological Diseases: Focus on Neuroprotection and Underlying Mechanisms. Front. Neurol..

[49] Rodríguez M., Valez V., Cimarra C., Blasina F., Radi R. (2020). Hypoxic-Ischemic Encephalopathy and Mitochondrial Dysfunction: Facts, Unknowns, and Challenges. Antioxid. Redox Signal..

[50] Zong Y., Li H., Liao P., Chen L., Pan Y., Zheng Y., Zhang C., Liu D., Zheng M., Gao J. (2024). Mitochondrial dysfunction: Mechanisms and advances in therapy. Signal Transduct. Target. Ther..

[51] Kim D.Y., Simeone K.A., Simeone T.A., Pandya J.D., Wilke J.C., Ahn Y., Geddes J.W., Sullivan P.G., Rho J.M. (2015). Ketone bodies mediate antiseizure effects through mitochondrial permeability transition. Ann. Neurol..

[52] Pawłowska M., Kruszka J., Porzych M., Garbarek J., Nuszkiewicz J. (2025). Ketogenic Metabolism in Neurodegenerative Diseases: Mechanisms of Action and Therapeutic Potential. Metabolites.

[53] Dardzinski B.J., Smith S.L., Towfighi J., Williams G.D., Vannucci R.C., Smith M.B. (2000). Increased plasma beta-hydroxybutyrate, preserved cerebral energy metabolism, and amelioration of brain damage during neonatal hypoxia ischemia with dexamethasone pretreatment. Pediatr. Res..

[54] Cai Y., Zhou W. (2025). Ferroptosis in Neonatal Hypoxic-Ischemic Encephalopathy: Mechanisms and the Therapeutic Potential of Vitamin D/VDR Signaling. Cell. Mol. Neurobiol..

[55] Burd I., Welling J., Kannan G., Johnston M.V. (2016). Excitotoxicity as a Common Mechanism for Fetal Neuronal Injury with Hypoxia and Intrauterine Inflammation. Adv. Pharmacol..

[56] Neves D., Salazar I.L., Almeida R.D., Silva R.M. (2023). Molecular mechanisms of ischemia and glutamate excitotoxicity. Life Sci..

[57] Kapur J. (2018). Role of NMDA receptors in the pathophysiology and treatment of status epilepticus. Epilepsia Open.

[58] Peerboom C., Wierenga C.J. (2021). The postnatal GABA shift: A developmental perspective. Neurosci. Biobehav. Rev..

[59] Falsaperla R., Collotta A.D., Sortino V., Malaventura C., Spatuzza M., Romano C., Suppiej A. (2025). The First 30 Days Postnatal of the GABA Receptor: A Comprehensive Overview. Mol. Neurobiol..

[60] Bjorkman S., Ireland Z., Colditz P., Miller S.M. (2010). Effect of Neonatal Hypoxia/Ischemia on Gabaa Receptor Protein Expression. Pediatr. Res..

[61] Cetinkaya M. (2024). Neuroprotective treatment options for neonatal hypoxic-ischemic encephalopathy: Therapeutic hypothermia and beyond. Glob. Pediatr..

[62] Chang P., Augustin K., Boddum K., Williams S., Sun M., Terschak J.A., Hardege J.D., Chen P.E., Walker M.C., Williams R.S. (2016). Seizure control by decanoic acid through direct AMPA receptor inhibition. Brain.

[63] Falsaperla R., Sortino V., Soler M.A., Spatuzza M., Fortuna S., Salpietro V. (2025). AMPA Receptor Modulation Through Medium-Chain Triglycerides and Decanoic Acid Supports Nutritional Intervention in Pediatric Epilepsy. Nutrients.

[64] Rogawski M.A., Löscher W., Rho J.M. (2016). Mechanisms of Action of Antiseizure Drugs and the Ketogenic Diet. Cold Spring Harb. Perspect. Med..

[65] Williams R.S.B., Boison D., Masino S.A., Rho J.M., Noebels J.L., Avoli M., Rogawski M.A., Vezzani A., Delgado-Escueta A.V. (2024). Mechanisms of Ketogenic Diet Action. Jasper′s Basic Mechanisms of the Epilepsies.

[66] Stuckey S.M., Ong L.K., Collins-Praino L.E., Turner R.J. (2021). Neuroinflammation as a Key Driver of Secondary Neurodegeneration Following Stroke?. Int. J. Mol. Sci..

[67] Zeng J., Bao T., Yang K., Zhu X., Wang S., Xiang W., Ge A., Zeng L., Ge J. (2023). The mechanism of microglia-mediated immune inflammation in ischemic stroke and the role of natural botanical components in regulating microglia: A review. Front. Immunol..

[68] Gaston-Breton R., Bouzid A., Antipushina E., Altaie A.M., Armengaud J., Costa N., Sarkadi B., Apati A., Harati R., Sharaev M. (2025). Translational biomarkers of hypoxic brain injury uncovered in CSF secreting human choroid plexus organoids. Fluids Barriers CNS.

[69] Ma Q. (2023). Pharmacological Inhibition of the NLRP3 Inflammasome: Structure, Molecular Activation, and Inhibitor-NLRP3 Interaction. Pharmacol. Rev..

[70] Xu S., Liu P., Jia J., Sun M., Ma F., Mao R., Li H., Ji S., Bao X., Xia S. (2025). Microglial NLRC5 drives lysosomal dysfunction to disrupt autophagic flux and promote post-stroke neuroinflammation. J. Neuroinflamm..

[71] Brochu M.E., Girard S., Lavoie K., Sébire G. (2011). Developmental regulation of the neuroinflammatory responses to LPS and/or hypoxia-ischemia between preterm and term neonates: An experimental study. J. Neuroinflamm..

[72] Li W., Liu E., Zhou Y., Liao Z., Wang D. (2025). Therapeutic potential of natural products in ischemic stroke: Targeting angiogenesis. Front. Pharmacol..

[73] Newman J.C., Verdin E. (2014). β-hydroxybutyrate: Much more than a metabolite. Diabetes Res. Clin. Pract..

[74] Zhou T., Cheng X., He Y., Xie Y., Xu F., Xu Y., Huang W. (2022). Function and mechanism of histone β-hydroxybutyrylation in health and disease. Front. Immunol..

[75] Ganai S.A., Ramadoss M., Mahadevan V. (2016). Histone Deacetylase (HDAC) Inhibitors—Emerging roles in neuronal memory, learning, synaptic plasticity and neural regeneration. Curr. Neuropharmacol..

[76] Moody L., Chen H., Pan Y.X. (2017). Early-Life Nutritional Programming of Cognition-The Fundamental Role of Epigenetic Mechanisms in Mediating the Relation between Early-Life Environment and Learning and Memory Process. Adv. Nutr..

[77] Shao R., Sun D., Hu Y., Cui D. (2021). White matter injury in the neonatal hypoxic-ischemic brain and potential therapies targeting microglia. J. Neurosci. Res..

[78] Back S.A., Rosenberg P.A. (2014). Pathophysiology of glia in perinatal white matter injury. Glia.

[79] Volpe J.J. (2009). Brain injury in premature infants: A complex amalgam of destructive and developmental disturbances. Lancet Neurol..

[80] Koper J.W., Lopes-Cardozo M., Van Golde L.M. (1981). Preferential utilization of ketone bodies for the synthesis of myelin cholesterol in vivo. Biochim. Biophys. Acta.

[81] Nehlig A. (2004). Brain uptake and metabolism of ketone bodies in animal models. Prostaglandins Leukot. Essent. Fat. Acids.

[82] Ari C., D′Agostino D.P., Cha B.J. (2024). Neuroregeneration Improved by Sodium-D,L-Beta-Hydroxybutyrate in Primary Neuronal Cultures. Pharmaceuticals.

[83] Back S.A., Han B.H., Luo N.L., Chricton C.A., Xanthoudakis S., Tam J., Arvin K.L., Holtzman D.M. (2002). Selective vulnerability of late oligodendrocyte progenitors to hypoxia-ischemia. J. Neurosci..

[84] Sampaio-Baptista C., Vallès A., Khrapitchev A.A., Akkermans G., Winkler A.M., Foxley S., Sibson N.R., Roberts M., Miller K., Diamond M.E. (2020). White matter structure and myelin-related gene expression alterations with experience in adult rats. Prog. Neurobiol..

[85] Gong Z., Faulkner M.E., Akhonda M.A.B.S., Guo A., Bae J., Laporte J.P., Church S., D′Agostino J., Bergeron J., Bergeron C.M. (2025). White matter integrity and motor function: A link between cerebral myelination and longitudinal changes in gait speed in aging. Geroscience.

[86] Lee B.L., Glass H.C. (2021). Cognitive outcomes in late childhood and adolescence of neonatal hypoxic-ischemic encephalopathy. Clin. Exp. Pediatr..

[87] Nehlig A., de Vasconcelos A.P. (1993). Glucose and ketone body utilization by the brain of neonatal rats. Prog. Neurobiol..

[88] Edmond J., Robbins R.A., Bergstrom J.D., Cole R.A., de Vellis J. (1987). Capacity for substrate utilization in oxidative metabolism by neurons, astrocytes, and oligodendrocytes from developing brain in primary culture. J. Neurosci. Res..

[89] Lu Y., Yang Y.Y., Zhou M.W., Liu N., Xing H.Y., Liu X.X., Li F. (2018). Ketogenic diet attenuates oxidative stress and inflammation after spinal cord injury by activating Nrf2 and suppressing the NF-κB signaling pathways. Neurosci. Lett..

[90] Hu Z.G., Wang H.D., Jin W., Yin H.X. (2009). Ketogenic diet reduces cytochrome c release and cellular apoptosis following traumatic brain injury in juvenile rats. Ann. Clin. Lab. Sci..

[91] Puchowicz M.A., Zechel J.L., Valerio J., Emancipator D.S., Xu K., Pundik S., LaManna J.C., Lust W.D. (2008). Neuroprotection in diet-induced ketotic rat brain after focal ischemia. J. Cereb. Blood Flow Metab..

[92] Prins M.L., Fujima L.S., Hovda D.A. (2005). Age-dependent reduction of cortical contusion volume by ketones after traumatic brain injury. J. Neurosci. Res..

[93] Noh H.S., Lee H.P., Kim D.W., Kang S.S., Cho G.J., Rho J.M., Choi W.S. (2004). A cDNA microarray analysis of gene expression profiles in rat hippocampus following a ketogenic diet. Mol. Brain Res..

[94] Zhao Z., Lange D.J., Voustianiouk A., MacGrogan D., Ho L., Suh J., Humala N., Thiyagarajan M., Wang J., Pasinetti G.M. (2006). A ketogenic diet as a potential novel therapeutic intervention in amyotrophic lateral sclerosis. BMC Neurosci..

[95] Odorcyk F.K., Duran-Carabali L.E., Rocha D.S., Sanches E.F., Martini A.P., Venturin G.T., Greggio S., da Costa J.C., Kucharski L.C., Zimmer E.R. (2020). Differential glucose and beta-hydroxybutyrate metabolism confers an intrinsic neuroprotection to the immature brain in a rat model of neonatal hypoxia ischemia. Exp. Neurol..

[96] Fabres R.B., Carvalho A.V.S., Silva Alós D.K.D., Machado D.N., Spies F.F., Cardoso D.S., Vido M.F.C., Martini A.P.R., Mattos M.M., Antunes B.P. (2025). Combined effects of β-hydroxybutyrate and therapeutic hypothermia in a neonatal hypoxia-ischemia model. J. Cereb. Blood Flow Metab..

[97] Lee B.S., Woo D.C., Woo C.W., Kim K.S. (2018). Exogenous β-Hydroxybutyrate Treatment and Neuroprotection in a Suckling Rat Model of Hypoxic-Ischemic Encephalopathy. Dev. Neurosci..

[98] Julio-Amilpas A., Montiel T., Soto-Tinoco E., Gerónimo-Olvera C., Massieu L. (2015). Protection of hypoglycemia-induced neuronal death by β-hydroxybutyrate involves the preservation of energy levels and decreased production of reactive oxygen species. J. Cereb. Blood Flow Metab..

[99] Zharikova A.A., Andrianova N.V., Silachev D.N., Nebogatikov V.O., Pevzner I.B., Makievskaya C.I., Zorova L.D., Maleev G.V., Baydakova G.V., Chistyakov D.V. (2025). Analysis of the brain transcriptome, microbiome and metabolome in ketogenic diet and experimental stroke. Brain Behav. Immun..

[100] Suzuki M., Suzuki M., Kitamura Y., Mori S., Sato K., Dohi S., Sato T., Matsuura A., Hiraide A. (2002). Beta-hydroxybutyrate, a cerebral function improving agent, protects rat brain against ischemic damage caused by permanent and transient focal cerebral ischemia. Jpn. J. Pharmacol..

[101] Falsaperla R., Sortino V., Collotta A.D., Privitera G.F., Palmeri A., Mauceri L., Ruggieri M. (2023). Ketogenic Diet in Neonates with Drug-Resistant Epilepsy: Efficacy and Side Effects-A Single Center's Initial Experience. Neuropediatrics.

[102] Thammongkol S., Vears D.F., Bicknell-Royle J., Nation J., Draffin K., Stewart K.G., Scheffer I.E., Mackay M.T. (2012). Efficacy of the ketogenic diet: Which epilepsies respond?. Epilepsia.

[103] Jagadish S., Payne E.T., Wong-Kisiel L., Nickels K.C., Eckert S., Wirrell E.C. (2019). The Ketogenic and Modified Atkins Diet Therapy for Children With Refractory Epilepsy of Genetic Etiology. Pediatr. Neurol..

[104] van der Louw E., van den Hurk D., Neal E., Leiendecker B., Fitzsimmon G., Dority L., Thompson L., Marchió M., Dudzińska M., Dressler A. (2016). Ketogenic diet guidelines for infants with refractory epilepsy. Eur. J. Paediatr. Neurol..

[105] Hsieh T.Y., Su T.Y., Hung K.Y., Hsu M.S., Lin Y.J., Kuo H.C., Hung P.L. (2023). Feasibility of ketogenic diet therapy variants for refractory epilepsy in neonates to infants under 2 years old. Epilepsy Behav..

[106] Dressler A., Trimmel-Schwahofer P., Reithofer E., Gröppel G., Mühlebner A., Samueli S., Grabner V., Abraham K., Benninger F., Feucht M. (2015). The ketogenic diet in infants--Advantages of early use. Epilepsy Res..

[107] Kossoff E.H., Turner Z., Bluml R.M., Pyzik P.L., Vining E.P. (2007). A randomized, crossover comparison of daily carbohydrate limits using the modified Atkins diet. Epilepsy Behav..

[108] Cobo N.H., Sankar R., Murata K.K., Sewak S.L., Kezele M.A., Matsumoto J.H. (2015). The ketogenic diet as broad-spectrum treatment for super-refractory pediatric status epilepticus: Challenges in implementation in the pediatric and neonatal intensive care units. J. Child Neurol..

[109] Villaluz M.M., Lomax L.B., Jadhav T., Cross J.H., Scheffer I.E. (2018). The ketogenic diet is effective for refractory epilepsy associated with acquired structural epileptic encephalopathy. Dev. Med. Child Neurol..

[110] Zhang H., Su S., Zhang H., Sun L., Liu Y., Liu G. (2024). Effectiveness and safety analysis of ketogenic diet therapy for drug-resistant epilepsy caused by structural pathology. Front. Neurol..

[111] Dou X., Xu X., Mo T., Chen H., Wang Z., Li X., Jia S., Wang D. (2022). Evaluation of the seizure control and the tolerability of ketogenic diet in Chinese children with structural drug-resistant epilepsy. Seizure.

